# Ant Colony System Optimization for Spatiotemporal Modelling of Combined EEG and MEG Data

**DOI:** 10.3390/e23030329

**Published:** 2021-03-11

**Authors:** Eugene A. Opoku, Syed Ejaz Ahmed, Yin Song, Farouk S. Nathoo

**Affiliations:** 1 Department of Mathematics and Statistics, University of Victoria, Victoria, BC V8P 5C2, Canada; yinsong@uvic.ca (Y.S.); nathoo@uvic.ca (F.S.N.); 2 Department of Mathematics and Statistics, Brock University, St. Catharines, ON L2S 3A1, Canada; sahmed5@brocku.ca

**Keywords:** ant colony system, bayesian spatial mixture model, inverse problem, nonparamteric boostrap, EEG/MEG data

## Abstract

Electroencephalography/Magnetoencephalography (EEG/MEG) source localization involves the estimation of neural activity inside the brain volume that underlies the EEG/MEG measures observed at the sensor array. In this paper, we consider a Bayesian finite spatial mixture model for source reconstruction and implement Ant Colony System (ACS) optimization coupled with Iterated Conditional Modes (ICM) for computing estimates of the neural source activity. Our approach is evaluated using simulation studies and a real data application in which we implement a nonparametric bootstrap for interval estimation. We demonstrate improved performance of the ACS-ICM algorithm as compared to existing methodology for the same spatiotemporal model.

## 1. Introduction

Electroencephalography (EEG) and Magnetoencephalography (MEG) are two non-invasive approaches for measuring electrical activity of the brain with high temporal resolution. These neuroimaging techniques allow us to study brain dynamics and the complex informational exchange processes in the human brain. They are widely used in many clinical and research applications [1,2], though estimating the evoked-response activity within the brain from electromagnetic fields measured outside of the skull remains a challenging inverse problem with infinitely many different sources within the brain that can produce the same observed data [3].

Proposed solutions to the MEG/EEG inverse problem have been based on distributed source and dipolar methods [4]. In the case of distributed source methods, every location on a fine grid within the brain has associated neural activation source parameters. In this case, the number of unknown current sources exceeds the number of MEG or EEG sensors and estimation thus requires constraints through regularization or priors to obtain a solution. For such methods, various steps have been taken to regularize the solution by choosing minimum-norm solutions or by limiting the spatiotemporal variation of the solution. These approaches impose $L_{2}$ or $L_{1}$ [5] norm regularization constraints that serve to stabilize and condition the source parameter estimates. However, these methods do not consider the temporal nature of the problem. In [6], the authors propose a dynamic state-space model that accounts for both spatial and temporal correlations within and across candidate intra-cortical sources using Bayesian estimation and Kalman filtering. Dipolar methods, on the other hand, assume that the actual current distribution can be explained by a small set of current dipoles with unknown locations, amplitudes and orientations (see [4] for review). Hence, the resulting inverse problem becomes non-linear and a number of dipoles is to be estimated. Proposed solutions to this problem include algorithms such as simulated annealing [7] to address nonlinear optimization in the localization of neuromagnetic sources.

From the perspective of Bayesian approaches, the ill-posed nature of the inverse problem requires incorporation of prior assumptions when choosing an appropriate solution out of an infinite set of candidates. For instance, the authors of [8] consider Gaussian scale mixture models, with flexible, large covariance components representing spatial patterns of neural activity. The authors of [9] also propose a hierarchical linear model with Gaussian errors in a Parametric Empirical Bayes (PEB) framework whose random terms are drawn from multivariate Gaussian distributions and covariances factor into temporal and spatial components at the sensor and source levels. The authors of [10] propose an application of empirical Bayes to the source reconstruction problem with automatic selection of multiple cortical sources. The authors of [11] developed the Mesostate-Space Model (MSM) based on the assumption that the unknown neural brain activity can be specified in terms of a set of locally distributed and temporally coherent meso-sources for either MEG or EEG data, while the authors of [12] extend this approach to propose a Switching Mesostate-Space Model (SMSM) to allow flexibility by accounting for complex brain processes that cannot be characterized by linear and stationary Gaussian dynamics.

By extending and building on the MSM, the authors of [13] developed a Bayesian spatial finite mixture model incorporating the following two conditions, taken directly from [13]:

1. Relaxing the assumption of independent mixture allocation variables and modeling mixture allocations using the Potts model, which allows for spatial dependence in allocations.
2. Formulate the model for combined MEG and EEG data for joint source localization.

This spatiotemporal model describes a joint model that combines MEG and EEG data, in which brain neural activity is modeled from the Gaussian spatial mixture model. The neural source activity is described in terms of a few hidden states, with each state having its own dynamics and a Potts model used in representing the spatial dependence in the mixture model.

For the Bayesian mixture model formulated, an Iterated Conditional Modes (ICM) algorithm was developed by the authors of [13] for simultaneous point estimation and model selection for the number of mixture components in the latent process. Whilst ICM is a very simple and computationally efficient algorithm, convergence of this algorithm is sensitive to starting values and local optima. This issue was left unresolved in [13]. Here we investigate the potential for finding better solutions, and focus on implementing a population-based optimization algorithm-based Ant Colony System (ACS) [14].

ACS is a metaheuristic optimization algorithm inspired by the biological behavior of ants constructing solutions based on their collective foraging behavior [14]. ACS has been successfully applied in several areas such as clustering, data mining and image segmentation problems [15,16,17]. ACS is a constructive algorithm that uses an analogue of ant trail pheromones to learn about good features of solutions in combinatorial optimization problems. New solutions are generated using a parameterized probabilistic model, the parameters of which are updated using previously generated solutions so as to direct the search towards promising areas of the solution space. The model used in ACS is known as pheromone, an artificial analogue of the chemical substance used by real ants to mark trails from the nest to food sources. Based on this representation, each artificial ant constructs a part of the solution based on concentration of pheromone information released by other ants. The amount of pheromone deposited by an ant reflects the quality of the good solutions built and the traversed path. The pheromone deposited and volatilized adds solution components to partial solutions. After some time and based on more ants’ communications through pheromone information, they tend to follow the same optimal paths yielding the optimal solution, in our context maximization of the posterior density.

As an alternative to the ICM algorithm, we thus implement the ACS algorithm coupled with a local search ICM algorithm to provide a new approach to model estimation and potentially better estimates of the model parameters. This approach is evaluated and found to provide significant improvements. Within the context of a simpler spatial mixture, ACS has been implemented for a Gaussian Potts mixture model in [18] and has been shown to outperform both the Simulated Annealing and ICM algorithms for parameter and mixture component estimation. The theoretical guarantees associated with simulated annealing to reach a global optimum is dependent on the choice of a cooling schedule. The choice of an optimal cooling schedule can be difficult in practice for large spatiotemporal models. ACS has also proved to be competitive with genetic and other optimization algorithms in several tasks, mainly in image classification and the traveling salesman problem [19,20].

Ant Colony Optimization (ACO) algorithms are implemented to solve Constraint Satisfaction Problems (CSP) where ACO solutions to CSP face the challenge of high cost and low solution quality. Motivated by this challenge, the authors of [21] proposed Ant Colony Optimization based on information Entropy (ACOE). The idea is based on incorporating a local search that uses a crossover operation to optimize the best solution according to the feedback of information entropy. This is performed by comparing the difference of the information entropy between the current global best solution and the best solution in the current iteration. Datasets from four classes of binary CSP test cases were generated and then ACOE was implemented for comparison. Results showed that ACOE outperformed Particle Swarm Optimization (PSO), a Differential Evolution (DE) algorithm and Artificial Bee Colony (ABC) in terms of the solution quality, data distribution and convergence performance.

To our knowledge, this is the first attempt at solving the neuroelectromagnetic inverse problem for combined EEG/MEG data using a population-based optimization approach combined with a spatial mixture model. The primary contribution of this paper is the design and implementation of the ACS algorithm to the dynamic spatial model and its evaluation. Importantly, we demonstrate improved results both in the estimation of neural activity and model selection uniformly across all conditions considered.

The rest of the paper proceeds as follows. The posterior distribution of the model and the design and implementation of the ACS algorithm are presented in Section 2. In Section 3 our algorithm is investigated using simulation studies and comparisons made with an existing approach developed in [13]. Section 4 provides an illustration on real data and the development of a nonparametric bootstrap for interval estimation in a study of scrambled face perception. The paper concludes with a conclusion and directions for future work in Section 4.

### Related Works

Merging EEG and MEG aims at accounting for information missed by one modality and captured by the other. Fused reconstruction therefore appears promising to reach high temporal and spatial resolutions in brain function imaging. The authors of [22] address the added value of combining EEG and MEG data for distributed source localization, building on the flexibility of parametric empirical Bayes, namely for EEG–MEG data fusion, group level inference and formal hypothesis testing. The proposed approach follows a two-step procedure by first using unimodal or multimodal inference to derive a cortical solution at the group level, and second by using this solution as a prior model for single subject-level inference based on either unimodal or multimodal data. Another popular approach for non-globally optimized solutions of the MEG/EEG inverse problem is based on the use of adaptive Beamformers (BF). However, the BFs are known to fail when dealing with correlated sources acting like poorly tuned spatial filters with a low signal-to-noise ratio (SNR) of the output time series and often meaningless cortical maps of power distribution. To address this limitation, the authors of [23] developed a novel data covariance approach to supply robustness to the beamforming technique when operating in an environment with correlated sources. To reduce the impact of the low spatial resolution of MEG and EEG, the authors of [24] developed a unifying framework for quantifying the spatial fidelity of MEG/EEG source estimates. This method quantifies the spatial fidelity of MEG/EEG estimates from simulated patch activations over the entire neocortex superposed on measured resting-state data. This approach grants more generalizability in the evaluation process that allows for, e.g., comparing linear and non-linear estimates in the whole brain for different Signal-to-Noise Ratios (SNR), number of active sources and activation waveforms. The authors of [25] discuss a solution to the source reconstruction problem and developed a novel hierarchical multiscale Bayesian algorithm for electromagnetic brain imaging using MEG and EEG within the context of sources that vary in spatial extent. In this Bayesian algorithm, the sensor data measurements are defined using a generative probabilistic graphical model that is hierarchical across spatial scales of brain regions and voxels. This algorithm enables robust reconstruction of sources that have different spatial extent, from spatially contiguous clusters of dipoles to isolated dipolar sources.

In [26], the authors propose a methodological framework for inverse-modeling of propagating cortical activity. Within this framework, cortical activity is represented in the spatial frequency domain, which is more natural than the dipole domain when dealing with spatially continuous activity. In dealing with multi-subject MEG/EEG source imaging, he authors of [27] propose a sparse multi-task regression that takes into account inter-subject variabilities known as the Minimum Wasserstein Estimates (MWE). This work jointly localizes sources for a population of subjects by casting the estimation as a multi-task regression problem in three key ideas. First, it proposes to use non-linear registration to obtain subject-specific lead field matrices that are spatially aligned. Second, it copes with the issue of inter-subject spatial variability of functional activations using optimal transport. Finally, it makes use of non-convex sparsity priors and joint inference of source estimates to obtain accurate source amplitudes. Various applications for MEG/EEG source reconstruction have been applied in the clinical setting for detection of epileptic spikes [28], identification of seizure onset zone [29] and presurgical workup of epilepsy patients [30,31,32].

## 2. Methods

This section describes the Bayesian spatial mixture model developed in [13] and the ACS-ICM algorithm.

### 2.1. Model

We provide details and mathematical description of the joint model below. Let $M\left(t\right)={\left(M_{1}\left(t\right),M_{2}\left(t\right),\ldots ,M_{n_{M}}\left(t\right)\right)}^{\prime }$ and $E\left(t\right)={\left(E_{1}\left(t\right),E_{2}\left(t\right),\ldots ,E_{n_{E}}\left(t\right)\right)}^{\prime }$ denote the MEG and EEG, respectively, at time *t*, t=1,…,T; where $n_{M}$ and $n_{E}$ denote the number of MEG and EEG sensors, the model assumes:

$$\begin{array}{cc} M(t) & =X_{M}S\left(t\right)+\epsilon_{M}\left(t\right),\;\;\;\;\;\;\epsilon_{M}\left(t\right)|\sigma_{M}^{2}\overset{iid}{∼}MVN\left(0,\sigma_{M}^{2}H_{M}\right),\;\;\;t=1,\ldots ,T,\\ E(t) & =X_{E}S\left(t\right)+\epsilon_{E}\left(t\right),\;\;\;\;\;\;\epsilon_{E}\left(t\right)|\sigma_{E}^{2}\overset{iid}{∼}MVN\left(0,\sigma_{E}^{2}H_{E}\right),\;\;\;t=1,\ldots ,T, \end{array}$$

where $X_{M}$ and $X_{E}$ denote $n_{M}\times P$ and $n_{E}\times P$ forward operators, respectively computed based on Maxwell’s equations under the quasi-static assumption [33] for EEG and MEG; $H_{M}$ and $H_{E}$ are known $n_{M}\times n_{M}$ and $n_{E}\times n_{E}$ matrices, respectively, which can be obtained from baseline data providing information on the covariance structure of EEG and MEG sensor noise; and $S\left(t\right)={\left(S_{1}\left(t\right),\ldots S_{P}\left(t\right)\right)}^{\prime }$ represents the magnitude and polarity of neural currents sources over a fine grid covering the cortical surface. In this case, *P* represents a large number of point sources of potential neural activity within the brain covering the cortical surface. It is assumed that the *P* cortical locations are embedded in a 3D regular grid composed of $N_{v}$ voxels to allow efficient computational implementation. Given this grid of voxels, a mapping $v:\left\{1,\ldots ,P\right\}\to \left\{1,\ldots ,N_{v}\right\}$ is defined such that v(j) is the index of the voxel containing the jth cortical location. We assume a latent Gaussian mixture with allocations at the level of voxels:

(1) $$S_{j}\left(t\right)|\mu \left(t\right),\alpha ,Z\overset{ind}{∼}\prod_{l=1}^{K}N{\left(\mu_{l}\left(t\right),\alpha_{l}\right)}^{Z_{v(j)l}},$$

j=1,…,P,t=1,…,T; where $Z={\left(Z_{1}^{{}^{\prime }},Z{}_{2}^{{}^{\prime }},\ldots ,Z{}_{N_{v}}^{{}^{\prime }}\right)}^{\prime }$ is a labeling process defined over the grid of voxels such that for each $v\in \{1,\ldots ,N_{v}\}$, $Z_{v}^{{}^{\prime }}=\left(Z_{v1},Z_{v2},\ldots Z_{vK}\right)$ with $Z_{vl}\in \left\{0,1\right\}$ and $\sum_{l=1}^{K}Z_{vl}=1$; $\mu \left(t\right)={\left(\mu_{1}\left(t\right),\mu_{2}\left(t\right),\ldots ,\mu_{K}\left(t\right)\right)}^{\prime }={\left(\mu_{1}\left(t\right),\mu^{A}{\left(t\right)}^{\prime }\right)}^{\prime }$, where $\mu^{A}\left(t\right)={\left(\mu_{2}\left(t\right),\ldots ,\mu_{K}\left(t\right)\right)}^{\prime }$ denotes the mean of the “active” states over different components of activity and $\mu_{1}\left(t\right)=0\;\;\mathrm{for}\;\mathrm{all}\;t$, so that the first component corresponds to an “inactive” state. The variability of the lth mixture component about its mean $\mu_{l}\left(t\right)$ is represented by $\alpha_{l},l=1,\ldots ,K$.

The labeling process assigns each voxel to a latent state and is assumed to follow a Potts model:

$$P\left(Z|\beta \right)=\frac{\exp\{\beta \sum_{h∼j}\delta \left(Z_{j},Z_{h}\right)\}}{G(\beta )},\;\;\;\;\;\delta \left(Z_{j},Z_{h}\right)=2Z_{j}^{{}^{\prime }}Z_{h}-1,$$

where G(β) is the normalizing constant for this probability mass function, β≥0 is a hyper-parameter that governs the strength of spatial cohesion, and i∼j indicates that voxels *i* and *j* are neighbors, with a first-order neighborhood structure over the 3D regular grid. The mean temporal dynamics for active components is assumed to follow a first-order vector autoregressive process:

$$\begin{array}{cc} \mu^{A}\left(t\right) & =A\mu^{A}\left(t-1\right)+a\left(t\right),\;\;\;a\left(t\right)|\sigma_{a}^{2}\overset{i.i.d}{∼}MVN\left(0,\sigma_{a}^{2}I\right) \end{array}$$

t=2,…,T, $\mu^{A}\left(1\right)∼MVN\left(0,\sigma_{\mu_{1}}^{2}I\right)$, with $\sigma_{\mu_{1}}^{2}$ fixed and known, but $\sigma_{a}^{2}$ unknown and assigned an inverse-Gamma ($a_{a},b_{a}$) hyper-prior. Although in [13] a pseudo-likelihood approximation is adopted to the normalizing constant of the Potts model and then assigned a uniform prior to the spatial parameter to control the degree of spatial correlation, we fixed the inverse temperature parameter and vary it as part of a sensitivity analysis.

For model selection, the number of mixture components, the value of *K*, in Equation (1) will not be known prior and so it is estimated simultaneously with model parameters. Thus this approach achieves simultaneous point estimation and model selection. We can obtain a simple estimate for the number of mixture components based on the estimated allocation variables $\hat{Z}$ when the algorithm is run with a sufficiently large value of *K*. This is achieved by running the algorithm with a value of *K* that is larger than the expected number of mixture components. For example, the value of *K* can be set as K=15 when running the algorithm. The jth location on the cortex is allocated to one of the mixture components based on the estimated value of ${\hat{Z}}_{v(j)}$, where ${\hat{Z}}_{v(j)}={\left({\hat{Z}}_{v{\left(j\right)}_{1}},{\hat{Z}}_{v{\left(j\right)}_{2}},\ldots ,{\hat{Z}}_{v{\left(j\right)}_{K}}\right)}^{\prime }$ and ${\hat{Z}}_{v{\left(j\right)}_{l}}=1$ if location *j* is allocated to component l∈{1,…,K}. When the algorithm is run with a value of *K* that is large, there will result empty mixture components that have not been assigned any voxel locations under $\hat{Z}$. In a sense these empty components have been automatically pruned out as redundant. The estimated number of mixture components can be obtained by counting the number of non-empty mixture components as follows:

$$\hat{K}=\sum_{l=1}^{K}I\left\{\sum_{v=1}^{n_{v}}{\hat{Z}}_{v_{l}}\neq 0\right\}.$$

This estimator requires us to run our algorithm only once for a single value of *K* and then the resulting number of mixture components assigned a location in $\hat{Z}$ is determined and $\hat{K}\leq K.$

### 2.2. Ant Colony System

Ant Colony System (ACS) is a population-based optimization algorithm introduced in [14]. The basic structure of this algorithm is designed to solve the traveling salesman problem in which the aim is to find the shortest path to cover a given set of cities without revisiting any one of them. The inspiring source and development of this algorithm is the observation of the foraging behavior of real ants in their colony. This behavior is exploited in artificial ant colonies for the search of approximate solutions to discrete optimization problems, for continuous optimization problems, and for important problems in telecommunications, such as routing and load balancing, telecommunication network design, or problems in bioinformatics [34,35]. At the core of this algorithm is the communication between the ants by means of chemical pheromone trails, which enables them to collectively find short paths between their nest and food source. The framework of this algorithm can be categorized into four main parts: (1) construction of an agent ant solution, (2) local pheromone update of the solution, (3) improving solution by local search, and (4) global pheromone update of the best solution.

At each step of this constructive algorithm a decision is made concerning which solution component to add to the sequence of solution components already built. These decisions are dependent on the pheromone information, which represents the learned experience of adding a particular solution component given the current state of the solution under construction. The accumulated amount of pheromone mirrors the quality of the solution constructed based on the value of the objective function. The pheromone update aims to concentrate the search in regions of the search space containing high quality solutions while there is a stochastic component facilitating random exploration of the search space. In particular, the reinforcement of solution components depending on the solution quality is an important ingredient of ACS algorithms. To learn which components contribute to good solutions can help assembling them into better solutions. In general, the ACS approach attempts to solve an optimization problem by iterating the following two steps: (1) candidate solutions are constructed using a pheromone model, that is, a parameterized probability distribution over the solution space; (2) the candidate solutions are used to modify the pheromone values in a way that is deemed to bias future sampling toward high quality solutions.

The posterior distribution of the dynamic model takes the form P(Θ|E,M) = P(Θ,E,M)/P(E,M), where:

(2) $$\begin{array}{cc} P(\Theta ,E,M) & =P(E,M|\Theta )P(\Theta )=P(E|\Theta )P(M|\Theta )P(\Theta )\\ \; & =\prod_{t=1}^{T}MVN\left(E\left(t\right);X{}_{E}S\left(t\right),\sigma_{E}^{2}H{}_{E}\right)\;\times MVN\left(M\left(t\right);X{}_{M}S\left(t\right),\sigma_{M}^{2}H{}_{M}\right)\\ \; & \times IG\left(\sigma_{E}^{2};a_{E},b_{E}\right)\times IG\left(\sigma_{M}^{2};a_{M},b_{M}\right)\times \left[\prod_{j=1}^{p}\prod_{t=1}^{T}\prod_{l=1}^{K}N{\left(S_{j}\left(t\right);\mu_{l}\left(t\right),\alpha_{l}\right)}^{Z_{v(j)l}}\right]\\ \; & \times \left[\prod_{t=2}^{T}MVN\left(\mu^{A}\left(t\right);A\mu^{A}\left(t-1\right),\sigma_{a}^{2}I\right)\right]\times MVN\left(\mu^{A}\left(1\right);0,\sigma_{\mu_{1}}^{2}I\right)\times \mathrm{Potts}\left(Z;\beta \right)\\ \; & \times \prod_{l=1}^{K}IG\left(\alpha_{l};a_{\alpha },b_{\alpha }\right)\times \times \left[\prod_{i=1}^{K-1}\prod_{j=1}^{K-1}N\left(A_{ij};0,\sigma_{A}^{2}\right)\right]\times IG\left(\sigma_{a}^{2};a_{a},b_{a}\right) \end{array}$$

where MVN(x;μ,Σ) denotes the density of the dim(x)-dimensional multivariate normal distribution with mean μ and covariance Σ evaluated at x; IG(x;a,b) denotes the density of the inverse gamma distribution with parameters *a* and *b* evaluated at *x*; $N(x;\mu ,\sigma^{2})$ denotes the density of the normal distribution with mean μ and variance $\sigma^{2}$ evaluated at *x*; Potts(Z;β) is the joint probability mass function of the Potts model with parameter β evaluated at Z. Equation (2) represents the objective function to be maximized over Θ. The goal is to optimize over $\Theta =\{S\left(t\right),Z,\mu \left(t\right),\alpha ,\sigma_{E}^{2},\sigma_{M}^{2},A,\sigma_{a}^{2}\}$ maximizing the posterior (2).

ACS is based on set of agents, each representing artificial ants that construct solutions as sequences of solution components. Agent ant *k* builds a solution by allocating label *ℓ* from a set of voxel labels Λ={1,…K} to the voxel $s\in \{1,\ldots ,N_{v}\}$ based on a probabilistic transition rule $p^{k}\left(s,\ell \right)$. The transition rule quantifies the probability of ant *k*, assigning voxel *s* to label *ℓ*. This transition rule depends on the pheromone information τ(s,ℓ) of the coupling (s,ℓ) representing the quality of assigning voxel *s* to label *ℓ* based on experience gathered by ants in the previous iteration. We let:

$$\ell =\left\{\begin{array}{cc} \arg\max_{u}\tau \left(s,u\right) & \mathrm{if}\;q\leq q_{o}\\ p^{k}\left(s,\ell \right) & \mathrm{if}\;q>q_{o} \end{array}\right.$$

(3) $$\begin{array}{c} p^{k}\left(s,\ell \right)=\frac{\tau (s,\ell )}{\sum_{u\in \Lambda }\tau \left(s,u\right)} \end{array}$$

where *ℓ* is a label for voxel *s* selected according to the transition rule above; q∼Uniform(0,1); $q_{o}\in \left(0,1\right)$ is a tuning parameter. An artificial ant chooses, with probability $q_{o}$, the solution component that maximizes the pheromone function τ(s,ℓ) or it performs, with probability $1-q_{o}$, a probabilistic construction step according to (3). The ACS pheromone system consists of two update rules; one rule is applied whilst constructing solutions (local pheromone update rule) and the other rule is applied after all ants have finished constructing a solution (global pheromone update rule). After assigning a label to a voxel, an ant modifies the amount of pheromone of the chosen couples (s,ℓ) by applying a local pheromone update (4):

(4) $$\begin{array}{c} \tau \left(s,\ell \right)\leftarrow \left(1-\rho \right)\tau \left(s,\ell \right)+\rho \tau_{o} \end{array}$$

where ρ∈(0,1) is a tuning parameter that controls evaporation of the pheromone and $\tau_{o}$ is the initial pheromone value. This operation simulates the natural process of pheromone evaporation preventing the algorithm from converging too quickly (all ants constructing the same solution) and getting trapped into a poor solution. In practice, the effect of this local pheromone update is to decrease the pheromone values via evaporation (1−ρ)τ(s,ℓ) on the visited solution components, making these components less desirable for the subsequents ants. The value of the evaporation rate indicates the relative importance of the pheromone values from one iteration to the following one. If ρ takes a value near 1, then the pheromone trail will not have a lasting effect, and this mechanism increases the random exploration of the search space within each iteration and helps avoid a too rapid convergence of the algorithm toward a sub-optimal region of the parameter space, whereas a small value will increase the importance of the pheromone, favoring the exploitation of the search space near the current solution.

To improve all solutions constructed and also update the other model parameters, we considered incorporating ICM as a local search method. Here, the ICM algorithm is used for both updating the model parameters and also for a local search over the mixture allocation variables. Thus, the update steps corresponding to ACS are combined with running ICM to convergence at each iteration. Finally, after all solutions have been constructed by combined ACS and ICM steps, the quality of all solutions is evaluated using the objective function where the corresponding best solution is selected. We use a global update rule, where pheromone evaporation is again applied on the best solution chosen. Assuming voxel *j* is assigned to label *v* for the best solution, the global update is given as:

$$\tau \left(j,v\right)\leftarrow \left\{\begin{array}{cc} \left(1-\rho \right)\tau \left(j,v\right)+\rho \tau_{o},\\ (1-\rho )\tau (j,k), & \mathrm{and}\;\mathrm{for}\;\mathrm{all}\;k\neq v \end{array}\right.$$

The steps described are performed repeatedly until a change in the objective function becomes negligible and the model parameters from the best solution are returned as the final parameter estimates. The optimal values for the tuning parameters $\left(q_{o},\tau_{o},\rho \right)$ used in our ACS-ICM algorithm depend on the data. The strategy we adopt for choosing the tuning parameters is by using an outer level optimization on top of the ACS-ICM algorithm to optimize over tuning parameters $\left(q_{o},\tau_{o},\rho \right)$ within updates at the outer level based on the Nelder–Mead algorithm [36] applied to optimize over tuning parameters.

In order to reduce the dimension of parameter space and computing time, we apply clustering to the estimated neural sources. This is achieved by implementing a K-means algorithm to cluster the *P* locations on the cortex into a smaller number of J≤P clusters, assuming that $S_{j}\left(t\right)=S_{l}\left(t\right)$ for cortical locations l,j belonging to the same cluster. We investigated different values of J=250,500,1000 in our simulation studies. Within the ICM algorithm, the labeling process Z is updated using an efficient chequerboard updating scheme [13]. The update scheme starts with partitioning Z into two blocks $Z=\{Z{}_{W},Z{}_{B}\}$ based on a three-dimensional chequerboard arrangement, where $Z{}_{W}$ corresponds to “white” voxels and $Z{}_{B}$ corresponds to “black” voxels. Under the Markov random field prior with a first-order neighborhood structure, the elements of $Z{}_{W}$ are conditionally independent given $Z{}_{B}$, the remaining parameters, and the data E, M. This allows us to update $Z{}_{W}$ in a single step, which involves simultaneously updating its elements from their full conditional distributions. The variables $Z{}_{B}$ are updated in the same way.

It is well-known that the ICM algorithm is sensitive to initial values and the authors of [13] found this to be the case with the ICM algorithm developed for the spatiotemporal mixture model. The solution obtained, and even the convergence of the algorithm depend rather heavily on the starting values chosen. In the case of ACS-ICM, regardless of the initial values, the algorithm finds a better solution with the optimal tuning parameters and this solution tends to be quite stable. This is because ACS-ICM is a stochastic search procedure in which the pheromone update concentrates the search in regions of the search space containing high quality solutions to reach an optimum. When considering a stochastic optimization algorithm, there are at least two possible types of convergence that can be considered: convergence in value and convergence in solution. With convergence in value, we are interested in evaluating the probability that the algorithm will generate an optimal solution at least once. On the contrary, with convergence in solution we are interested in evaluating the probability that the algorithm reaches a state that keeps generating the same optimal solution. The convergence proofs are presented in [37,38]. The authors of [37] proved convergence with a probability of 1−ϵ for the optimal solution and more in general for any optimal solution in [38] of the ACS algorithm. This supports the argument that theoretically the application of ACS-ICM to source reconstruction should improve ICM.

The local search ICM algorithm procedure is presented in Algorithm 1 and the ACS-ICM algorithm is presented in Algorithm 2. Convergence of the ICM algorithms is monitored by examining the relative change of the Frobenius norm of the estimated neural sources on consecutive iterations.

Algorithm 1 presents a detailed description of the ICM algorithm. The ICM algorithm requires full conditional distributions of each model parameter where the mode of the distribution is taken as the update step for the parameter. The full conditional distribution are described and presented in [13]. This ICM algorithm is embedded in our ACS-ICM algorithm.
**Algorithm 1** Iterated Conditional Modes (ICM) Algorithm.1:$\Theta =\{S\left(t\right),Z,\mu \left(t\right),\alpha ,\sigma_{E}^{2},\sigma_{M}^{2},A,\sigma_{a}^{2}\}\leftarrow \mathrm{Initial}\;\mathrm{Value}$    2:Converged←0    3:**while**Converged=0**do**4:    $\sigma_{M}^{2}\leftarrow \left[\sum_{t=1}^{T}\frac{1}{2}{\left(M\left(t\right)-X{}_{M}S\left(t\right)\right)}^{\prime }H{}_{M}^{-1}\left(M\left(t\right)-X{}_{M}S\left(t\right)\right)+b_{M}\right]/\left[a_{M}+\frac{TN_{M}}{2}+1\right]$    5:    $\sigma_{E}^{2}\leftarrow \left[\sum_{t=1}^{T}\frac{1}{2}{\left(E\left(t\right)-X{}_{E}S\left(t\right)\right)}^{\prime }H{}_{E}^{-1}\left(E\left(t\right)-X{}_{E}S\left(t\right)\right)+b_{E}\right]/\left[a_{E}+\frac{TN_{E}}{2}+1\right]$6:    $\sigma_{a}^{2}\leftarrow \left[\sum_{t=2}^{T}\frac{1}{2}{\left(\mu^{A}\left(t\right)-A\mu^{A}\left(t-1\right)\right)}^{\prime }\left(\mu^{A}\left(t\right)-A\mu^{A}\left(t-1\right)\right)+b_{a}\right]/\left[a_{a}+\frac{\left(T-1)(K-1\right)}{2}+1\right]$    7:    $vec\left(A\right)\leftarrow {\left(\frac{1}{\sigma_{a}^{2}}\left(\sum_{t=2}^{T}\mu^{A}{\left(t\right)}^{\prime }{\mathrm{Kr}}_{t}\right)\times C_{1}^{-1}\right)}^{\prime }$, where $C{}_{1}=\frac{1}{\sigma_{A}^{2}}I_{{\left(K-1\right)}^{2}}+\frac{1}{\sigma_{a}^{2}}\left(\sum_{t=2}^{T}{\mathrm{Kr}}_{t}^{\prime }{\mathrm{Kr}}_{t}\right)$,and ${\mathrm{Kr}}_{t}=\left(\mu^{A}{\left(t-1\right)}^{\prime }⊗I_{K-1}\right)$   8:    **for** l=1,...,K **do**    9:        $\alpha_{l}\leftarrow [\left[\frac{\sum_{j=1}^{P}\sum_{t=1}^{T}Z_{v(j)l}{\left(S_{j}\left(t\right)-\mu_{l}\left(t\right)\right)}^{2}}{2}+b_{\alpha }\right]/\left[\frac{T\sum_{j=1}^{P}Z_{v(j)l}}{2}+a_{\alpha }+1\right]$10:    **end for**11:    $\mu \left(1\right)\leftarrow {\left(\left(\sum_{j=1}^{P}{\left(S_{j}\left(1\right){\vec{I}}_{K-1}\right)}^{\prime }D_{j}+\frac{1}{\sigma_{a}^{2}}\mu^{A}{\left(2\right)}^{\prime }A\right)\times B_{1}^{-1}\right)}^{\prime }$, where $B_{1}=\sum_{j=1}^{P}D_{j}+\frac{1}{\sigma_{a}^{2}}A^{\prime }A+\frac{1}{\sigma_{\mu_{1}}^{2}}I_{K-1},\;D_{j}=\mathrm{Diag}\left(\frac{Z_{v(j)l}}{\alpha_{l}},l=2,\ldots ,K\right),\;{\vec{I}}_{K-1}={\left(1,1,\ldots ,1\right)}^{\prime }\;\mathrm{with}\;\dim\;\left({\vec{I}}_{K-1}\right)=K-1$    12:    **for** t=2,...,T−1 **do**    13:        $\mu \left(t\right)\leftarrow {\left(\left(\sum_{j=1}^{P}{\left(S_{j}\left(t\right){\vec{I}}_{K-1}\right)}^{\prime }D_{j}+\frac{1}{\sigma_{a}^{2}}{\left(\mu^{A}\left(t+1\right)\right)}^{\prime }A+\frac{1}{\sigma_{a}^{2}}\left(\mu^{A}{\left(t-1\right)}^{\prime }A^{\prime }\right)\right)\times B_{2}^{-1}\right)}^{\prime }$where $B_{2}=\sum_{j=1}^{P}D_{j}+\frac{1}{\sigma_{a}^{2}}\left(A^{\prime }A+I_{K-1}\right)$14:    **end for**15:    $\mu \left(T\right)\leftarrow {\left(\left(\sum_{j=1}^{P}{\left(S_{j}\left(T\right){\vec{I}}_{K-1}\right)}^{\prime }D_{j}+\frac{1}{\sigma_{a}^{2}}\left(\mu^{A}{\left(T-1\right)}^{\prime }A^{\prime }\right)\right)\times B_{3}^{-1}\right)}^{\prime }$where $B_{3}=\sum_{j=1}^{P}D_{j}+\frac{1}{\sigma_{a}^{2}}I_{k-1}$    16:    **for** j=1,...,P **do**17:        $S_{j}\leftarrow -\frac{1}{2}\Sigma_{S_{j}}W_{2j}$            ▹$S_{j}={\left(S_{j}\left(1\right),S_{j}\left(2\right),\ldots ,S_{j}\left(T\right)\right)}^{\prime }$$\Sigma_{S_{j}}^{-1}=W_{1j}I{}_{T}$, $W_{2j}^{\prime }=\left(W_{2j}\left(1\right),W_{2j}\left(2\right),\ldots ,W_{2j}\left(T\right)\right)$where $W_{1j}=\frac{1}{\sigma_{M}^{2}}\left(X{}_{M}{\left[,j\right]}^{\prime }H{}_{M}^{-1}X{}_{M}\left[,j\right]\right)+\frac{1}{\sigma_{E}^{2}}\left(X{}_{E}{\left[,j\right]}^{\prime }H{}_{E}^{-1}X{}_{E}\left[,j\right]\right)+\sum_{l=1}^{K}\frac{Z_{v(j)l}}{\alpha_{l}}$    $W_{2j}\left(t\right)=\frac{1}{\sigma_{M}^{2}}\left(-2M{\left(t\right)}^{\prime }H{}_{M}^{-1}X{}_{M}\left[,j\right]+2{\left(\sum_{v\neq j}^{}X{}_{M}\left[,v\right]S_{v}\left(t\right)\right)}^{\prime }H{}_{M}^{-1}X{}_{M}\left[,j\right]\right)\;+\frac{1}{\sigma_{E}^{2}}\left(-2E{\left(t\right)}^{\prime }H{}_{E}^{-1}X{}_{E}\left[,j\right]+2{\left(\sum_{v\neq j}^{}X{}_{E}\left[,v\right]S_{v}\left(t\right)\right)}^{\prime }H{}_{E}^{-1}X{}_{E}\left[,j\right]\right)-2\sum_{l=1}^{K}\frac{\mu_{l}\left(t\right)}{\alpha_{l}}$    $X{}_{M}\left[,j\right],\;X{}_{E}\left[,j\right]$ denote the jth column of $X{}_{E}$ and $X{}_{M}$    18:    **end for**19:    Let B denote the indices for “black” voxels and W denote the indices for “white” voxels.    20:    **for** κ∈Bsimultaneously **do**    21:        $Z_{\kappa q}\leftarrow 1$ and $Z_{\kappa l}\leftarrow 0,\;\;\forall l\neq q$where $q={\mathrm{argmax}}_{h\in \{1,\ldots ,K\}}P\left(h\right)$, and    22:        $P\left(h\right)=\frac{\alpha_{h}^{-TN_{j\kappa }/2}\times \exp\left(-\frac{1}{2}\sum_{j|v(j)=\kappa }\alpha_{h}^{-1}\sum_{t=1}^{T}{\left(S_{j}\left(t\right)-\mu_{h}\left(t\right)\right)}^{2}+2\beta \sum_{v\in \delta_{\kappa }}Z_{vh}\right)}{\sum_{l=1}^{K}\alpha_{l}^{-TN_{j\kappa }/2}\times \exp\left(-\frac{1}{2}\sum_{j|v(j)=\kappa }\alpha_{l}^{-1}\sum_{t=1}^{T}{\left(S_{j}\left(t\right)-\mu_{l}\left(t\right)\right)}^{2}+2\beta \sum_{v\in \delta_{\kappa }}Z_{vl}\right)}$    where $N_{j\kappa }$ is the number of cortical locations contained in voxel κ.    23:    **end for**24:    **for** κ∈Wsimultaneously **do**25:        $Z_{\kappa q}\leftarrow 1$ and $Z_{\kappa l}\leftarrow 0,\;\;\forall l\neq q$where $q={\mathrm{argmax}}_{h\in \{1,\ldots ,K\}}P\left(h\right)$, and    26:        $P\left(h\right)=\frac{\alpha_{h}^{-TN_{j\kappa }/2}\times \exp\left(-\frac{1}{2}\sum_{j|v(j)=\kappa }\alpha_{h}^{-1}\sum_{t=1}^{T}{\left(S_{j}\left(t\right)-\mu_{h}\left(t\right)\right)}^{2}+2\beta \sum_{v\in \delta_{\kappa }}Z_{vh}\right)}{\sum_{l=1}^{K}\alpha_{l}^{-TN_{j\kappa }/2}\times \exp\left(-\frac{1}{2}\sum_{j|v(j)=\kappa }\alpha_{l}^{-1}\sum_{t=1}^{T}{\left(S_{j}\left(t\right)-\mu_{l}\left(t\right)\right)}^{2}+2\beta \sum_{v\in \delta_{\kappa }}Z_{vl}\right)}$    where $N_{j\kappa }$ is the number of cortical locations contained in voxel κ.    27:    **end for**   28:    Check for convergence. Set Converged = 1 if so.    29:**end while**

**Algorithm 2** Ant Colony System (ACS)-ICM Algorithm.
1:Θ↢ Initial Value; set tuning parameters $\tau_{o}$, $q_{o}$, ρ and $N_{ants}$.    2:Initialize pheromone information $\tau \left(i,\ell \right)=\tau_{o}$, for each $\left(i,\ell \right)\in \left\{1,\ldots N_{v}\right\}\times \left\{1,\ldots K\right\}$ representing information gathered by ants.    3:Construct candidate solutions for each of $N_{ants}$ ants. For ant *j*, we find a candidate voxel labeling $Z^{\left(j\right)}={\left(Z_{1}^{{{}^{\prime }}^{\left(j\right)}},Z_{2}^{{{}^{\prime }}^{\left(j\right)}},\ldots ,Z_{N_{v}}^{{{}^{\prime }}^{\left(j\right)}}\right)}^{\prime }.$ This is done sequentially for each ant *j*.Construct candidate by assigning label *l* to voxel *s* using the transition probability rule:
$$\ell =\left\{\begin{array}{cc} \arg\max_{u}\tau \left(s,u\right) & \mathrm{if}\;q\leq q_{o}\\ p(s,\ell ) & \mathrm{if}\;q>q_{o} \end{array}\right.$$
where if $q>q_{o}$ the label for voxel *s* is drawn randomly from {1,…,K} with probability
$$p\left(s,\ell \right)=\frac{\tau (s,\ell )}{\sum_{u\in \Lambda }\tau \left(s,u\right)},$$
and where q∼uniform[0,1].    Assuming voxel *s* is assigned label *ℓ* set:
$$\tau \left(s,\ell \right)\leftarrow \left(1-\rho \right)\tau \left(s,\ell \right)+\rho \tau_{o}$$
and for all k≠l
τ(s,ℓ)←(1−ρ)τ(s,k)
where ρ is a tuning parameter in (0,1), which represents evaporation of the pheromone trails and $\tau_{o}>0$.4:For all ants, improve candidate solutions by running ICM to convergence (this also allows an update to the other model parameters) $\Theta =\{\left\{\mu^{A}\left(1\right),\mu^{A}\left(2\right),\ldots ,\mu^{A}\left(T\right)\right\},\left\{\alpha_{1},\alpha_{2},\ldots ,\alpha_{k}\right\},\sigma_{E}^{2},\sigma_{M}^{2},\left\{S_{j}\left(t\right),t=1,2\ldots ,T,j=1,2,\ldots ,P\right\},A,\sigma_{a}^{2}\}.$5:For all $N_{ants}$ solutions, evaluate the quality of each ant’s solution using objective function: P(Θ,E,M). Keep track of the best value. The current solution for each ant serves as the starting value for the next iteration.   6:Apply global updating of the pheromone function. For the best solution, (s,ℓ) update the pheromone as follows:Assuming voxel *s* is assigned label *ℓ* set:
$$\tau \left(s,\ell \right)\leftarrow \left(1-\rho \right)\tau \left(s,\ell \right)+\rho \tau_{o}$$
and for all k≠ℓ:
τ(s,ℓ)←(1−ρ)τ(s,k)Check for convergence via increase in logP(Θ,E,M). Go back to step 3    7:Return all voxel labeling Z and model parameters Θ from the best solution.


## 3. Simulation Studies

In this section, we use a simulation study to evaluate the performance of our algorithm. The simulation study assesses the quality of the source estimates and the optimized objective function values obtained when using our proposed algorithm in comparison to the existing ICM algorithm developed in [13]. We then make comparisons between ACS-ICM and the ICM algorithm applied to combined simulated EEG and MEG data.

### 3.1. Proposed Approach

**Figure A1:**
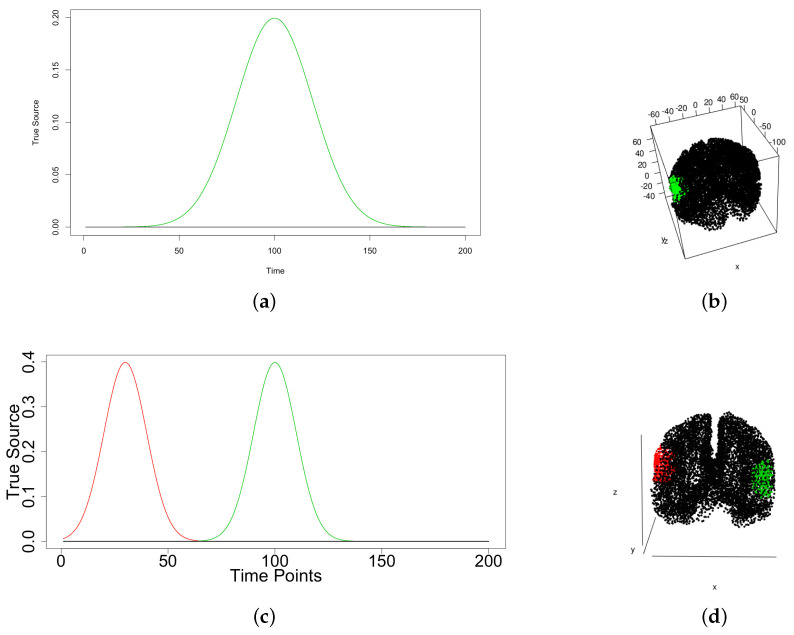

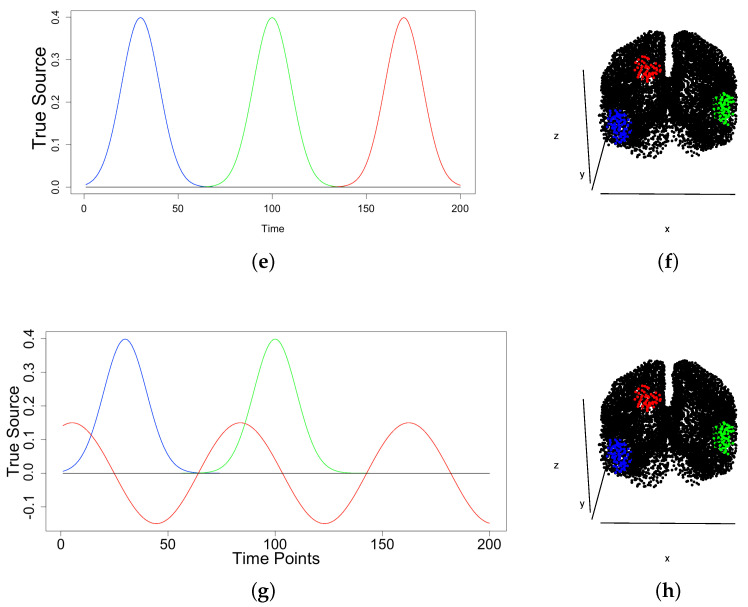
The true signal $S_{j}\left(t\right)$ used in each of the distinct active and inactive regions in the simulation studies of Section 3 for K=2; panel (**a**), K=3; panel (**c**) and K=4; panel (**e**,**g**) are depicted in the left column. The right column presents the true partition of the cortex into active and inactive states for the corresponding states for K=2; panel (**b**), K=3; panel (**d**) and K=4; panel (**f**,**h**).

**Figure A2:**
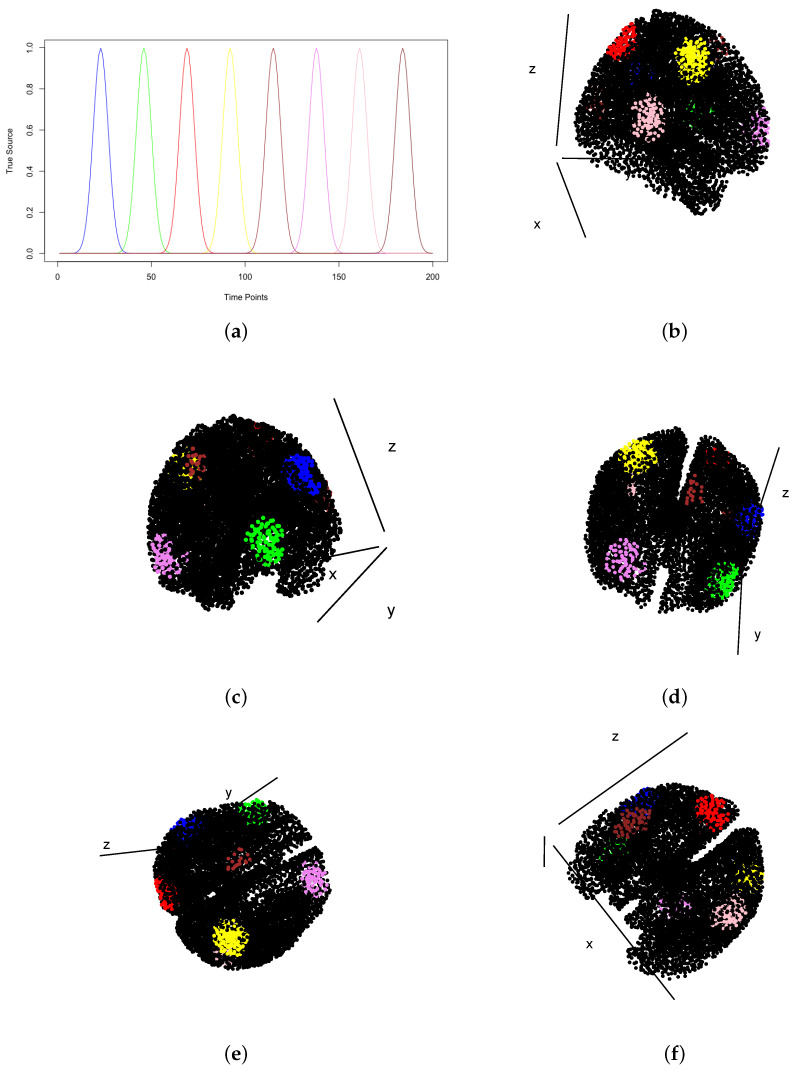
The true signal $S_{j}\left(t\right)$ in panel (**a**) and true partition of the cortex into active and inactive states for the case of K=9 states (panel **b**–**f**) used in simulation studies of Section 3.

The MEG and EEG data were both generated from four scenarios with two, three, four and nine latent states corresponding to regions of neural activity. In each of the four cases, one of the states is inactive, while the remaining states represent different regions of brain activity generated by Gaussian signals. The temporal profile of brain activity at each of the brain locations in the activated regions is depicted in Appendix A, Figure A1 and Figure A2. We projected the source activity at 8196 brain locations from the cortex onto the MEG and EEG sensor arrays using the forward operators $X_{M}$ and $X{}_{E}$. The simulated data were then obtained by adding Gaussian noise at each sensor, where the variance of the noise at each sensor was set to be 5% of the temporal variance of the signal at that sensor. The number of mixture components *K* was set to be the true number of latent states (either two, three, four, or nine) in the model. We simulated 500 replicate datasets and both ACS-ICM and ICM were applied to each dataset. For each simulated dataset we applied our algorithm with J = 250, 500, 1000 clusters so as to evaluate how the performance varies as this tuning parameter changes. We initialized both algorithms using the same starting values. For each replicate we computed the correlation between the estimated sources and the true sources $\mathrm{Corr}[\left(S{\left(1\right)}^{\prime },S{\left(2\right)}^{\prime },\ldots ,S{\left(T\right)}^{\prime }\right),\left(\hat{S}{\left(1\right)}^{\prime },\hat{S}{\left(2\right)}^{\prime },\ldots ,\hat{S}{\left(T\right)}^{\prime }\right)]$ as a measure of agreement. This measure was also averaged over the 500 replicate datasets to compute average correlation. In addition, we estimated the Mean-Squared Error (MSE) of the estimator ${\hat{S}}_{j}\left(t\right)$ based on the R = 500 simulation replicates for each brain location *j* and time point *t*. The Total MSE (TMSE) was computed by adding all the MSE’s over brain locations and time points. This was done separately for locations in active and inactive regions.

In our simulation studies, the ACS-ICM algorithm had four tuning parameters. The first denoted as $q_{o}\in \left(0,1\right)$ controlled the degree of stochasticity, with larger values corresponding to less stochasticity and thus less random exploration of the parameter space. When a solution is chosen, another tuning parameter $\tau_{0}$ controlled the amount of pheromone reinforcing this solution in the information available to the other ants. A third tuning parameter ρ controlled the evaporation of pheromone, and finally a fourth tuning parameter $N_{ants}$ controlled the number of ants. The number of ants $\left(N_{ants}\right)$ was fixed at 10, a value for which we have seen generally good performance. This was chosen based on computing efficiency and similar results (objective function values) from using $N_{ants}\geq 10$. The remaining optimal tuning parameters $\left(q_{o},\tau_{0},\rho \right)$ for all simulations cases were chosen using an outer level optimization using the Nelder–Mead algorithm.

### 3.2. Simulation Results

#### 3.2.1. Evaluation of Neural Source Estimation

We present the average correlation between the estimated values and the truth for the algorithms considered in our study in Appendix A, Table A1. Inspecting Table A1, we observe that for all cases considered for the true number of latent states (either two, three, four, or nine), the estimates obtained from the ACS-ICM algorithm yielded a higher average correlation than those obtained from ICM. In addition, with respect to the number of clusters, ACS-ICM resulted in a higher average correlation than ICM uniformly for all cluster sizes (250, 500, 1000). In summary, the average correlation was significantly improved when estimates were computed using the ACS-ICM algorithm for both large and small numbers of latent states as well as cluster sizes. In addition, we present in Appendix A, Figure A4, violin plots comparing the correlation values obtained from each of the algorithms for different simulation cases across all replicates. These plots show the entire distribution and provide a better assessment of each algorithm for simulation replicates. Observing Figure A4, we can see that ACS-ICM provides the highest correlation values uniformly in all simulation scenarios.

The TMSE for all simulation scenarios is presented in Appendix A, Table A2. To improve the readability of the results from TMSE values, we computed the relative percentage improvement in TMSE of the neural source estimators from ICM to ACS-ICM. Here, using ICM as the reference algorithm, the relative percentage improvement is defined as the ratio of the difference in TMSE between ICM and ACS-ICM to its ICM TMSE value multiplied by 100. The results of this computation are presented in Table 1. In all simulation scenarios for Table 1, ACS-ICM performed better and showed a significant improvement as compared to ICM. Specifically with respect to the number of clusters, ACS-ICM was roughly 10% better than ICM with respect to TMSE when the cluster size was 250. For both small and large numbers of latent states, ACS-ICM was better than ICM in the active region with significant improvements. This shows that ACS-ICM outperforms ICM in active regions using both small and large numbers of latent states. The total MSEs were decomposed into total variance and total squared bias for the same distinct cases of the simulation depicted in Table 1. From the results, when we considered active regions with different numbers of clusters, and observed that ACS-ICM was better than ICM based on the total squared bias due to the percentage of relative change. Based on the total variance we also noticed a similar positive change from ICM to ACS-ICM uniformly for all values of K. It is also clear that for inactive regions, ACS-ICM was better than ICM for both total variance and squared bias for all simulation cases considered. Overall, these results from the TMSE demonstrate a significant improvement obtained from our algorithm when considering total squared bias and variance for our simulation studies. This improvement was observed uniformly across all conditions.

**Figure 1:**
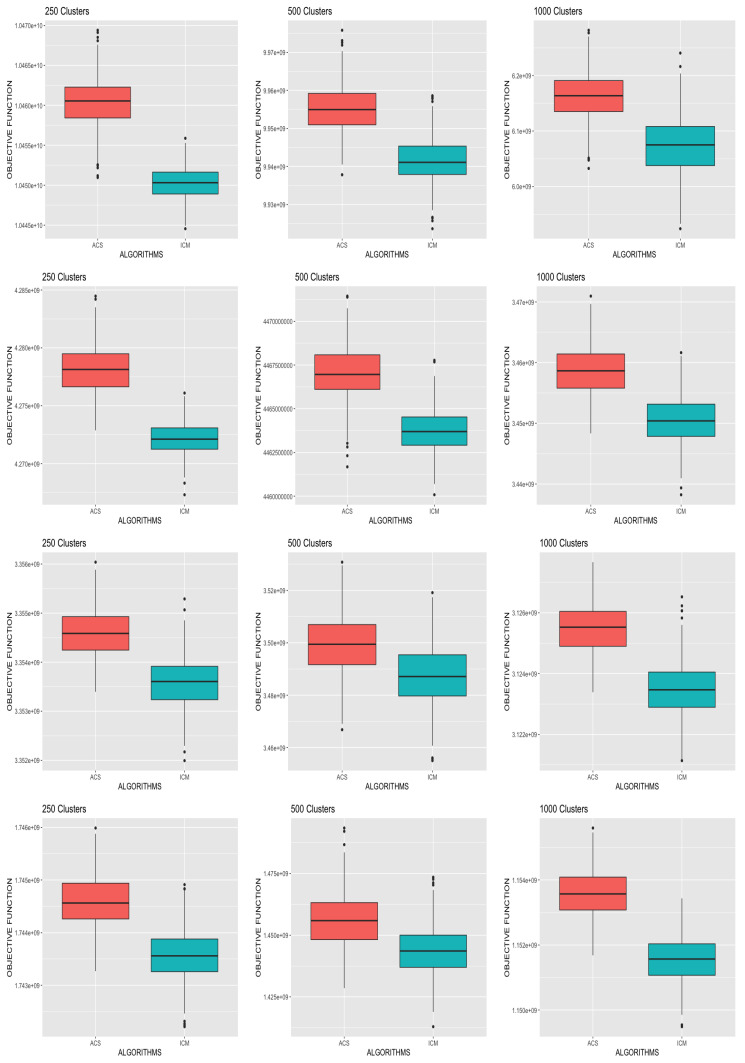
Box-plots comparing the objective function values obtained in the simulation studies for the ICM and ACS-ICM algorithms. The first row corresponds to the case when K=2, second row corresponds to when K=3, third row is when K=4 and the last row is when K=9.

We present in Figure 1 boxplots comparing the final objective function values obtained from each of the algorithms for the different simulation scenarios across all replicates. Again, a clear pattern emerged showing that ACS-ICM yielded the highest objective function values uniformly in all cases. Overall, ACS-ICM outperformed the ICM algorithm uniformly with respect to both neural source estimates and the values of the objective function. This indicates the superiority of the ACS-ICM algorithm over ICM for computing neural source estimates for the spatiotemporal model.

#### 3.2.2. Evaluation of Mixture Component Estimation

In addition to evaluating point estimation and objective function maximization, we also evaluated model selection, comparing ${\hat{K}}_{ACS}$ and ${\hat{K}}_{ICM}$, that is, the estimators obtained from ACS-ICM and ICM, respectively. We focused on estimating the number of mixture components and evaluating the sampling distribution of ${\hat{K}}_{ACS}$ and ${\hat{K}}_{ICM}$. The following five scenarios were considered in our experiments:

1. Two latent states with Gaussian source activity in the active regions depicted in Appendix A, Figure A1, panel (a).
2. Three latent states with Gaussian source activity in the active regions depicted in Appendix A, Figure A1, panel (c).
3. Four latent states with Gaussian source activity in the active regions depicted in Appendix A, Figure A1, panel (e).
4. Four latent states with Gaussian and sinusoidal source activity in the active regions depicted in Appendix A, Figure A1, panel (g).
5. Nine latent states with Gaussian source activity in the active regions depicted in Appendix A, Figure A2, panel (a).

**Figure 2:**
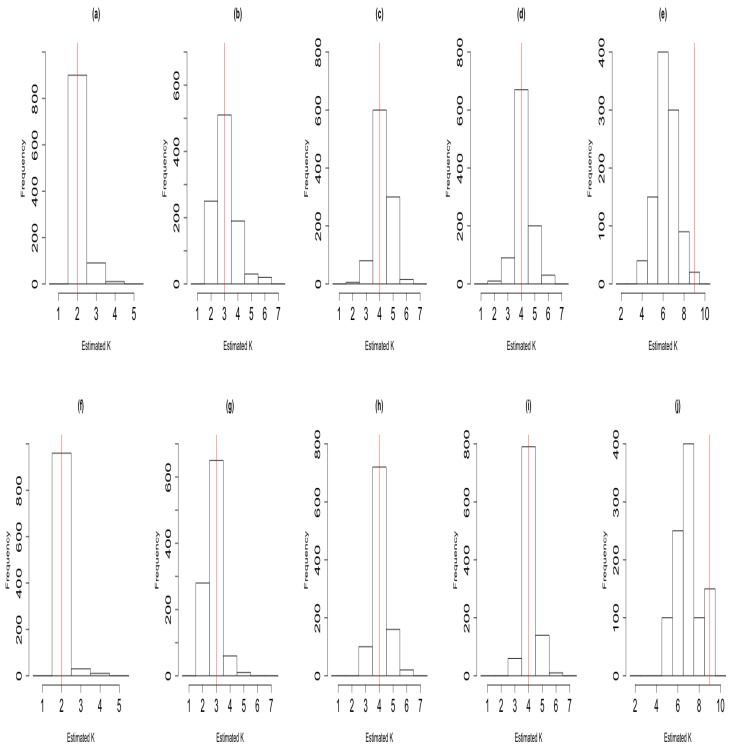
Histograms illustrating the sampling distribution of $\hat{K}$ in the case where the true signals were well separated in the simulation studies. The first row corresponds to the sampling distribution of ${\hat{K}}_{ICM}$; panel (**a**), K=2; panel (**b**), K=3; panel (**c**), K=4 with three Gaussian signals; panel (**d**), K=4 with two Gaussian signals and one sinusoid; panel (**e**), K=9 with eight Gaussian signals. The second row corresponds to the sampling distribution of ${\hat{K}}_{ACS}$; panel (**f**), K=2; panel (**g**), K=3; panel (**h**), K=4 with three Gaussian signals; panel (**i**), K=4 with two Gaussian signals and one sinusoid; panel (**j**), K=9 with eight Gaussian signals. In each case the vertical red line indicates the true number of latent states underlying the simulated data.

We simulated the data for each of the five scenarios considered, and added 5% Gaussian noise at the sensors with 1000 replicate datasets used in each case. The algorithms were run with an upper bound of K=10 for each of the 5000 simulated datasets. For each dataset, we computed the value of the estimator, and histograms representing the sampling distributions are presented in Figure 2, for each of the five cases above illustrating the sampling distribution of ${\hat{K}}_{ICM}$ (panels (a)–(e)) and ${\hat{K}}_{ACS}$ (panels (f)–(j)) corresponding to the first and second row, respectively. Observing Figure 2, where the true signals are well separated in the simulation experiments, in all cases except for the case with a larger number of latent states (K=9), the mode of the sampling distributions corresponds to the true number of latent states for both the ACS-ICM and ICM algorithms. In the case of nine neural sources, ACS-ICM gave better and improved results than ICM. Additionally, Table 2 reports both the bias and mean-squared error of the estimators from ACS-ICM (${\hat{K}}_{ACS}$) and ICM (${\hat{K}}_{ICM}$). From Table 2, both ACS-ICM and ICM are biased and over-estimated for the small number of latent states but underestimated for the large number of latent states. More importantly, the estimate for the number of mixture components obtained from ACS-ICM exhibited the best performance in terms of both bias and MSE uniformly for all cases considered. This is based on $|\mathrm{Bias}\left({\hat{K}}_{ACS}\right)|<|\mathrm{Bias}\left({\hat{K}}_{ICM}\right)|$ and $\mathrm{MSE}\left({\hat{K}}_{ACS}\right)<\mathrm{MSE}\left({\hat{K}}_{ICM}\right)$.

**Figure 3:**
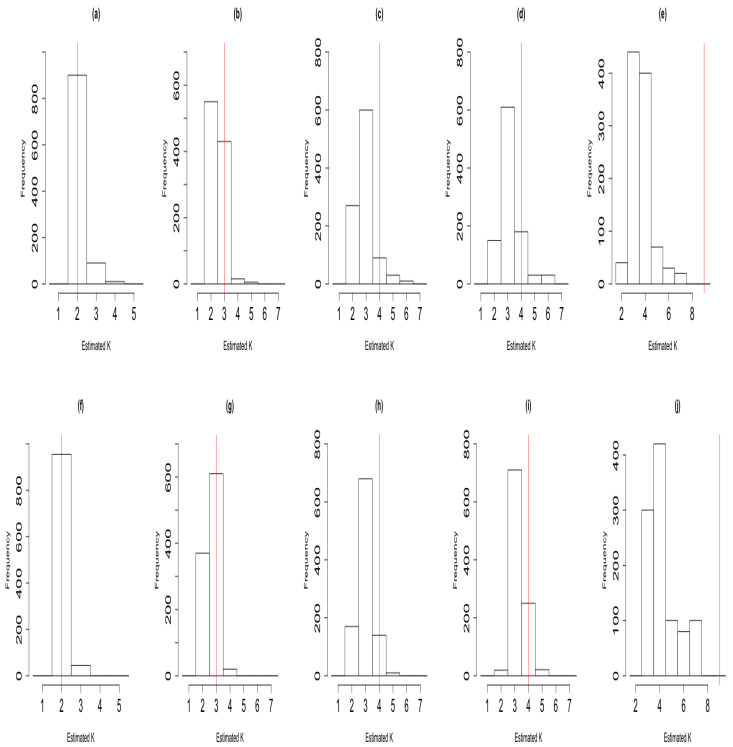
Histograms illustrating the sampling distribution of $\hat{K}$ in the case where the true signals were less well-separated in the simulation studies. The first row corresponds to the sampling distribution of ${\hat{K}}_{ICM}$; panel (**a**), K=2; panel (**b**), K=3; panel (**c**), K=4 with three Gaussian signals; panel (**d**), K=4 with two Gaussian signals and one sinusoid; panel (**e**), K=9 with eight Gaussian signals. The second row corresponds to the sampling distribution of ${\hat{K}}_{ACS}$; panel (**f**), K=2; panel (**g**), K=3; panel (**h**), K=4 with three Gaussian signals; panel (**i**), K=4 with two Gaussian signals and one sinusoid; panel (**j**), K=9 with eight Gaussian signals. In each case the vertical red line indicates the true number of latent states underlying the simulated data.

**Figure A3:**
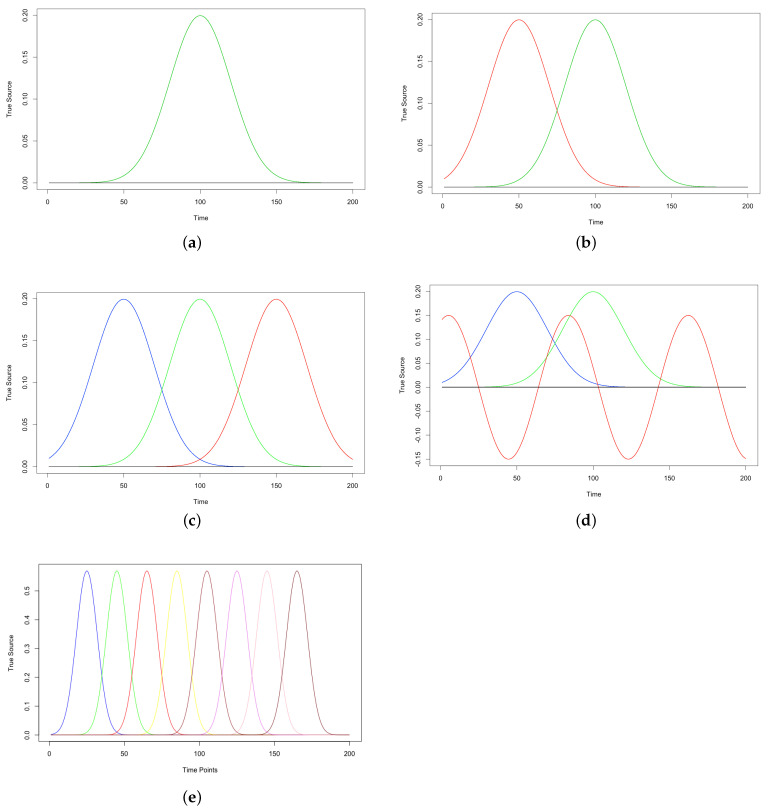
The true signal $S_{j}\left(t\right)$ used in in each of the distinct active and inactive regions for K=2; panel (**a**), K=3; panel (**b**), K=4; panel (**c**,**d**), K=9; panel (**e**) in the simulation study of Section 3.2, in the second part of the study where the mixture components were less well separated.

**Figure A4:**
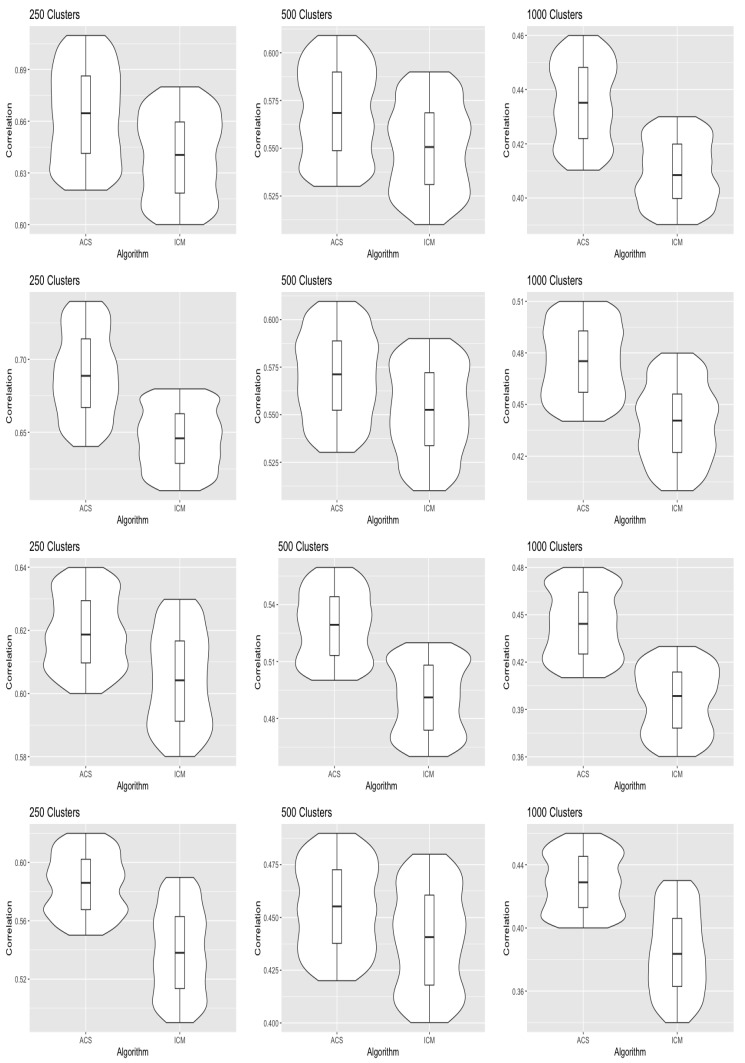
Violin plots comparing the correlation values obtained in the simulation studies for the ICM and ACS-ICM algorithms. The first row corresponds to the case when K=2, the second row corresponds to when K=3, the third row is when K=4 and the last row is when K=9.

We repeated the simulation studies for all five cases but where true signals are less well separated by altering the true signals depicted in Appendix A, Figure A3. We present histograms depicted in Figure 3, for each of the five cases above, illustrating the sampling distribution of ${\hat{K}}_{ICM}$ (panels (a)–(e)) and ${\hat{K}}_{ACS}$ (panels (f)–(j)). In this case, the mode of the sampling distribution corresponds to the true number of latent states when K=2 and K=3 but not for the case with four and nine latent states with both algorithms. In Table 2 we compare the bias and mean square error of ${\hat{K}}_{ICM}$ and ${\hat{K}}_{ACS}$ under this simulation settings. Similarly, under these settings, ACS-ICM outperformed ICM in terms of the bias and mean square error; thus, $|\mathrm{Bias}\left({\hat{K}}_{ACS}\right)|<|\mathrm{Bias}\left({\hat{K}}_{ICM}\right)|$ and $\mathrm{MSE}\left({\hat{K}}_{ACS}\right)<\mathrm{MSE}\left({\hat{K}}_{ICM}\right)$. In summary, for model selection, based on the results presented in Table 2, ACS-ICM showed an overall better performance over ICM uniformly for all eight conditions considered.

Whereas the ACS-ICM algorithm showed superiority in terms of quality of source estimates, a drawback is that it is computationally expensive relative to ICM due to its population-based and iterative procedure. Notwithstanding, this might not be a serious challenge for source localization problems, which do not require real-time solutions in most situations. With regards to computation time, on the Niagara cluster running R software on a single core (Intel Skylake 2.4 GHz, AVX512), ICM computed source estimates in approximately 2 min whereas ACS-ICM computed estimates in roughly 6 h and 30 min.

## 4. Application to Scrambled Face Perception MEG/EEG Data

**Figure 4:**
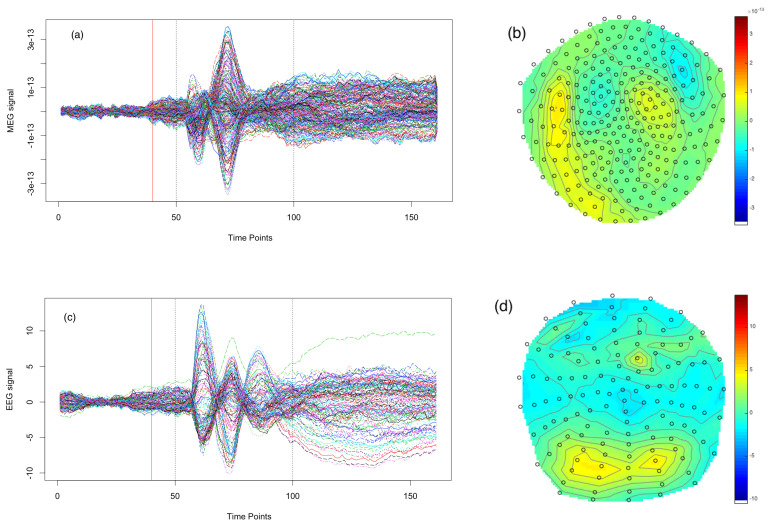
The Magnetoencephalography (MEG) and Electroencephalography (EEG) data considered in the face perception study: panels (**a**,**c**) show the time series observed at each MEG sensor and EEG sensor, respectively; panels (**b**,**d**) depict the spatially interpolated values of the MEG data and the EEG data, respectively, each observed at t=80, roughly 200 ms after presentation of the stimulus. In panels (**b**,**d**) the black circles correspond to the sensor locations after projecting these locations onto a 2-dimensional grid (for presentation).

In this section, we present the application of our methodology for comparison with EEG and MEG data measuring an event-related response to the visual presentation of scrambled faces in a face perception study. In addition, we demonstrate how a nonparametric bootstrap can be used to obtain standard errors, confidence intervals and T-maps. The data from both MEG and EEG were obtained from a single subject in an experimental paradigm that involved repeated random presentation of a picture showing either a face or a scrambled face while the subject was required to make a symmetry judgement. The scrambled faces were created through 2D Fourier transformation, random phase permutation, inverse transformation and outline-masking of each face. The experiment involved a sequence of trials, each lasting 1800 ms, where in each trial the subject was presented with one of the pictures for a period of 600 ms while being required to make a four-way, left–right symmetry judgment while brain activity was recorded over the array. Both scrambled faces and unscrambled faces were presented to the subject; however, our analysis will focus only on trials involving scrambled faces. This produced a multivariate time series for each trial, and the trial-specific time series were then averaged across trials to create a single multivariate time series; the average evoked response is depicted in Figure 4, panel (a), for MEG data, and panel (c), for EEG data. Looking from a spatial perspective, at a given time point, each array recorded a spatial field such as that depicted in Figure 4, panel (b), which shows the MEG spatial field at a particular time point, and Figure 4, panel (d), which shows the EEG spatial field at the same time point. The degree of inter-trial variability was quite low. This experiment was conducted while EEG data were recorded, and then again on the same subject while MEG data were recorded.

The EEG data were acquired on a 128-sensors ActiveTwo system with a high sampling rate of 2048 Hz and down-sampled to 200 Hz. The EEG data were re-referenced to the average over good channels. The resulting EEG data were a trial-specific multivariate time series and contained 128 sensors, 161 time points and 344 trials. For real data analysis, the trial-specific time series were averaged across trials to produce a single average evoked response. The MEG data were acquired on 274 sensors with a CTF/VSM system, with a high sampling rate of 480 Hz and down-sampled to 200 Hz. The MEG data obtained were a trial -specific multivariate time series and contained 274 sensors, 161 time points and 336 trials. We obtained a temporal segment of the data from time point t = 50 to t = 100, resulting in 51 time points for both the EEG and MEG data. The trial-specific time series were averaged across trials to produce a single average evoked response. Detailed description of the data and related analysis can be found in [9,39,40]. In addition, a link to the open access data repository used for analysis can be found here: https://www.fil.ion.ucl.ac.uk/spm/data/mmfaces (accessed on 14 November 2020).

**Figure 5:**
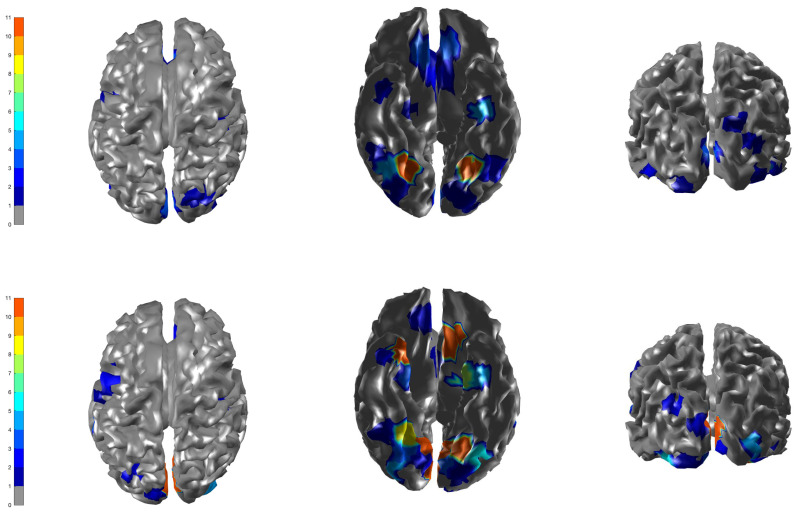
Brain activation for scrambled faces—the power of the estimated source activity $\sum_{t=1}^{T}{\hat{S}}_{j}{\left(t\right)}^{2}$ at each location *j* of the cortical surface. **Row 1** displays results from our ICM algorithm applied to the combined MEG and EEG data; **Row 2** displays results from ACS-ICM applied to the combined MEG and EEG data.

**Figure 6:**
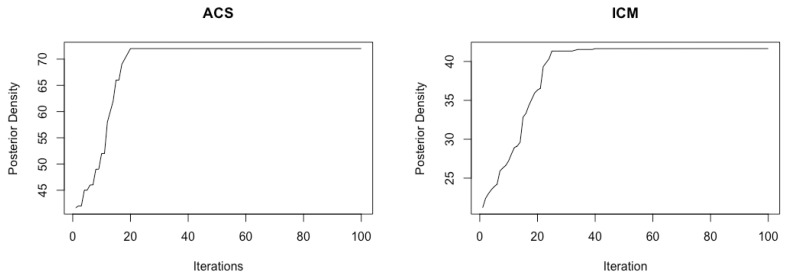
Objective function values obtained from the data with the ACS-ICM (**left**) and ICM (**right**) algorithms.

We set the upper bound at K=10 mixture components, voxels as $n_{v}$ = 560, β = 0.3 (hyperparameter of Potts model) and a cluster size of J = 250. For our real data application, the optimal tuning parameters $\left(q_{o},\tau_{0},\rho ,N_{ants}\right)=\left(0.43,0.05,0.64,10\right)$ were selected similarly using the Nelder–Mead algorithm. First, the ICM algorithm was run to convergence and the estimates obtained were used as the initial values for the ACS-ICM algorithm. Our primary interest lies in the estimated neural sources $\hat{S}\left(t\right)$ and we computed the total power of these estimated sources obtained from both algorithms at each brain location, which was then mapped onto the cortex. The cortical maps showing the spatial patterns from the estimated power of the reconstructed sources are displayed in Figure 5. The first and second row depict the corresponding results obtained from the ICM and ACS-ICM algorithms, respectively. As shown in Figure 5, the greatest power occurred on the bilateral ventral occipital cortex for both estimated sources from the ACS-ICM and ICM algorithms. Interestingly, the results from ACS-ICM estimates also differed when compared with the results from ICM in the left ventral frontal and right ventral temporal regions. In particular, the ACS-ICM estimate detected higher power, whereas ICM showed low activation in these regions. The estimated source locations of these region is responsible for high-level visual processing. Therefore, the cortical power map seems to represent regions that would be expected to show scrambled face-related activity. To compare the general quality of the estimates from ACS-ICM versus ICM, we show the plot of the final objective function values obtained from the algorithms in Figure 6. We see clearly that the application of ACS-ICM has led to higher quality estimates with much larger posterior density values.

**Figure 7:**
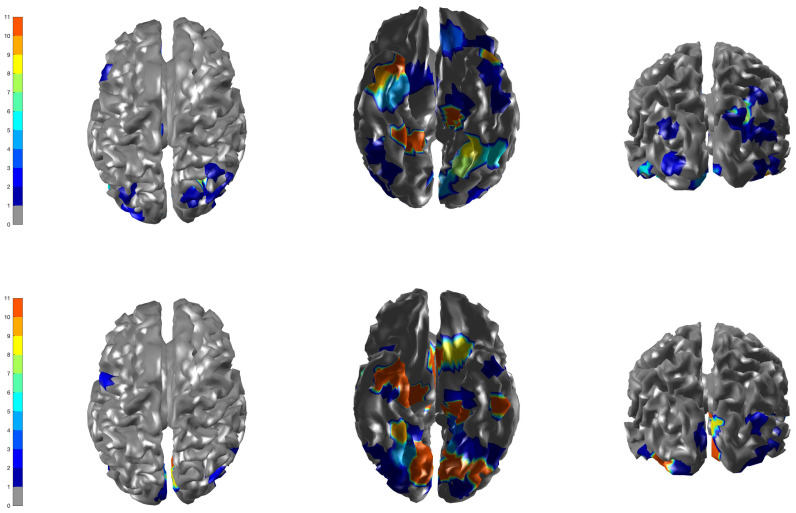
The spatial profile of brain activity from ACS-ICM based on our bootstrap replicates. **Row 1** displays standard deviations of the total power of the estimated source activity; **Row 2** displays the T-map.

The ACS-ICM algorithm used to maximize the posterior distribution produces only point estimates of the neural source activity. In addition to the point estimates, we applied a nonparametric bootstrap on the trial-specific multivariate time series to obtain confidence interval estimates and characterize the variability in our source estimates, which is another extension to [13]. The interval estimates were constructed by resampling the trial-specific MEG/EEG time series data with replacement. From each resampled dataset, we obtained the average evoked response and then run the ACS-ICM algorithm for a total of 400 nonparametric bootstrap replicates. This procedure was made feasible using parallel computation on a large number of computing cores. We constructed a cortical map of the bootstrap standard deviations of the total power of the estimated source. To account for uncertainty in our point estimates, we constructed a T-map and this is depicted in Figure 7. A T-map is the ratio of the ACS-ICM point estimate of the source activity to its bootstrap standard deviations. The T-map represents the best depiction of reconstructed power since it accounts for standard errors that a simple map of the point estimates does not. Broadly, the T-map seems to indicate similar results to those obtained from point estimates, in particular with respect to high power activation on the bilateral ventral occipital cortex and right ventral temporal region. An interesting observation from the T-map is the detection of a high signal in the left ventral temporal region but a low activation from the point estimate.

**Figure 8:**
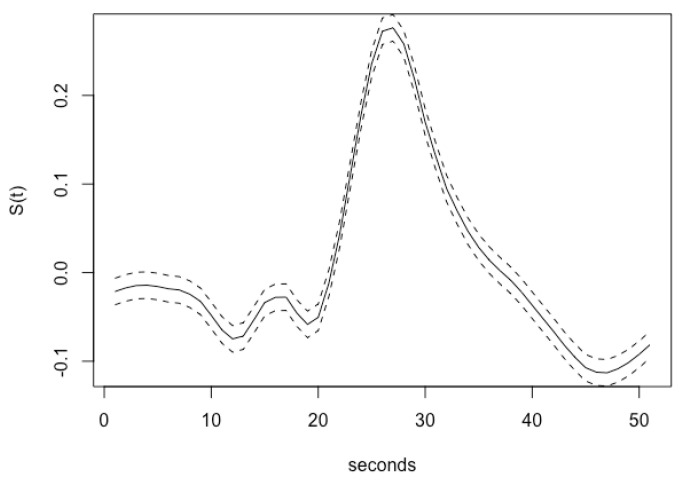
The 95% confidence interval for the estimated temporal profile of brain activity at the peak location of the T-map from the bootstrap replicates.

**Figure 9:**
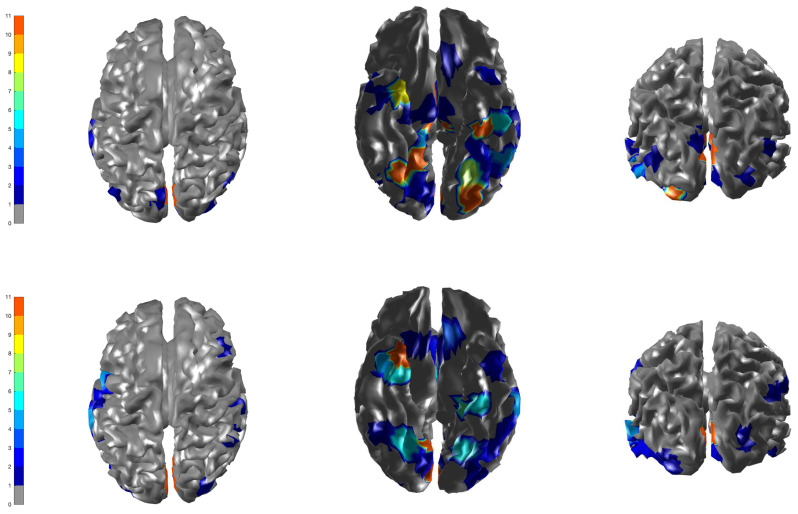
Brain activation for scrambled faces—the power of the estimated source activity $\sum_{t=1}^{T}{\hat{S}}_{j}{\left(t\right)}^{2}$ at each location *j* of the cortical surface. **Row 1** displays results from our ACS-ICM algorithm applied to the combined MEG and EEG data with β=0.1; **Row 2** displays results from ACS-ICM applied to the combined MEG and EEG data with β=0.44.

In addition, we present the temporal summary from our bootstrap replicates representing the interval estimation for the estimated temporal profile of brain activity at the peak location of the T-map. The interval estimate represents a 95% confidence interval depicted in Figure 8. One of the key components of our work is varying the inverse temperature parameter for sensitivity analysis. We fixed the inverse temperature at β=(0.1,0.44) and run the ACS-ICM algorithm to convergence. We run our algorithm together with K=10, $n_{v}$ = 560 and a cluster size of J = 250. For β=0.1, the corresponding results obtained are depicted in the first row of Figure 9. The results indicate activation on the bilateral ventral occipital cortex. Additionally, at β=0.44, the power map results from ACS-ICM, depicted in the second row of Figure 9, differ when compared with results from ACS-ICM at β=0.1 In particular, the highest power signals occured in the right ventral temporal region where there was low activation for using β=0.1.

**Figure A5:**
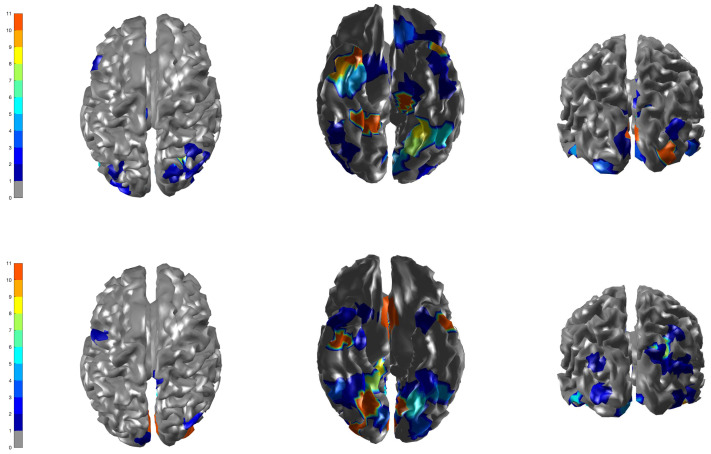
Brain activation for scrambled faces—the power of the estimated source activity $\sum_{t=1}^{T}{\hat{S}}_{j}{\left(t\right)}^{2}$ at each location *j* of the cortical surface. **Row 1** displays results from our ICM algorithm applied to the combined MEG and EEG data; **Row 2** displays results from ACS applied to the combined MEG and EEG data.

**Figure A6:**
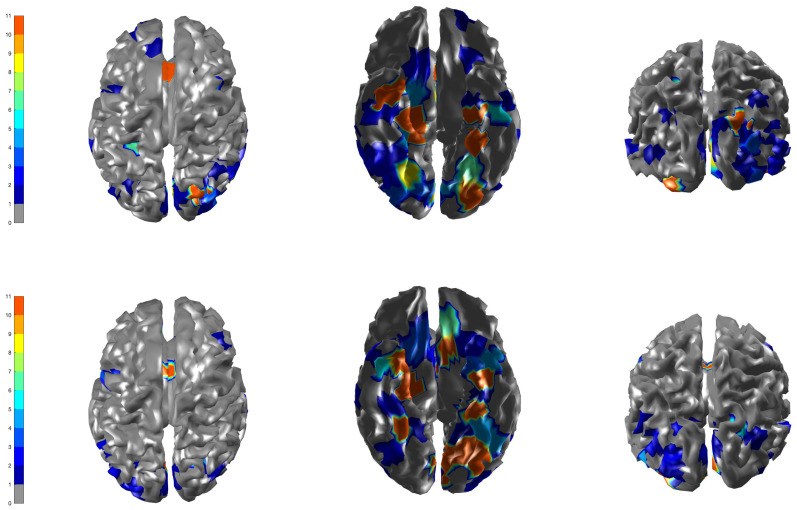
Brain activation for scrambled faces—the power of the estimated source activity $\sum_{t=1}^{T}{\hat{S}}_{j}{\left(t\right)}^{2}$ at each location *j* of the cortical surface. **Row 1** displays results from our ACS applied to the combined MEG and EEG data with β=0.1; **Row 2** displays results from ACS applied to the combined MEG and EEG data with β=0.44.

For our real data application we applied both algorithms with J = 500 clusters so as to evaluate how the performance varies as this tuning parameter changes. The results are displayed in Appendix B. The corresponding results obtained from ACS-ICM are displayed in the second row of Figure A5. Examining Figure A5, ACS-ICM seems to indicate similar results to those obtained from using a tuning parameter of J = 250, in particular with respect to activation on the bilateral ventral occipital cortex. For our sensitivity analysis, we present results obtained from using inverse temperature (β=0.1 and β=0.44) displayed in Figure A6. We observe that from ACS-ICM, the spatial spread of the high power occurs on the bilateral ventral occipital cortex. In addition, source estimates obtained from ACS-ICM indicate bilateral activation in the occipital cortex, and activation in the right temporal and right frontal regions of the brain. These estimated source locations reveal activation in areas known to be involved in the processing of visual stimuli. More interestingly, ACS-ICM also detected high power in a region on the corpus callosum; given that the inverse problem is ill-posed with an infinite number of possible configurations this may be the reason.

In our real data analysis, the required computation time for ICM was 3 min on a single core (Intel Skylake 2.4 GHz, AVX512) with R software, whereas the computation time for the ACS-ICM was roughly 7 h. The choice of cluster size will have an impact on the computational time required by the algorithm. With regards to ACS-ICM, the required computing time for a cluster size of 250 was approximately 7 h, whereas for a cluster size of 500, ACS-ICM required 12 h of computing time. While there is a substantially increase in computation, the paper has demonstrated uniform improvements in the quality of the solutions, in terms of both source estimation and model selection. Furthermore, the bootstrap can be implemented in parallel on a computing cluster to obtain standard errors with no increase to the required computation time.

### Residual Diagnostics for the Scrambled Faces MEG and EEG Data

**Figure 10:**
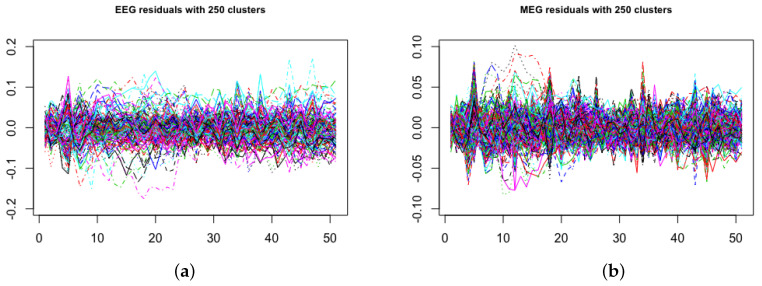

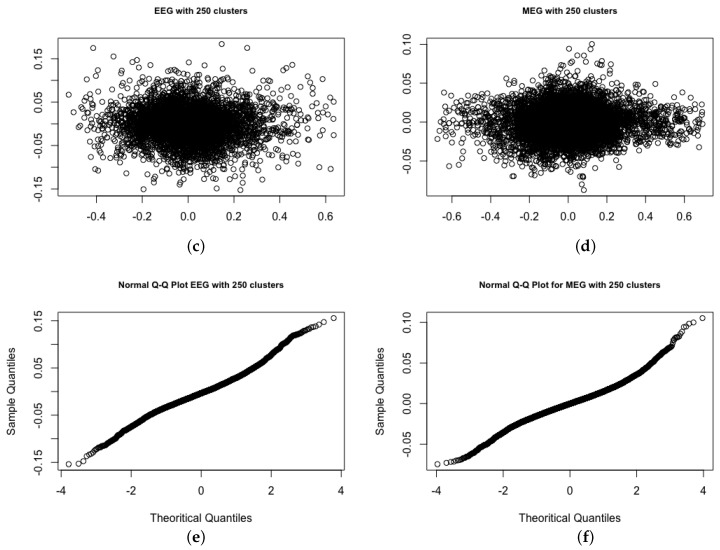
Brain activation for scrambled faces using the ACS-ICM algorithm—residual diagnostics: time series of residuals, (**a**) EEG, (**b**) MEG; residuals versus fitted values, (**c**) EEG, (**d**) MEG; residual normal quantile–quantile plots, (**e**) EEG, (**f**) MEG.

**Figure 11:**
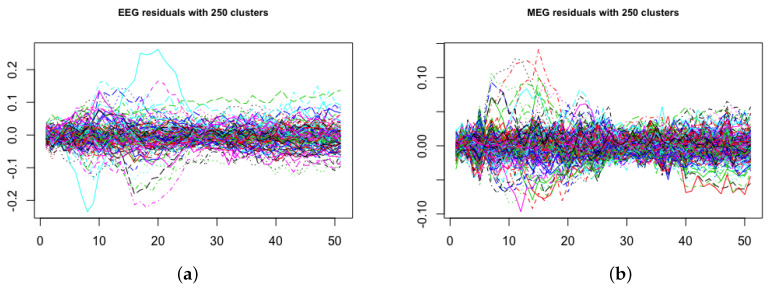

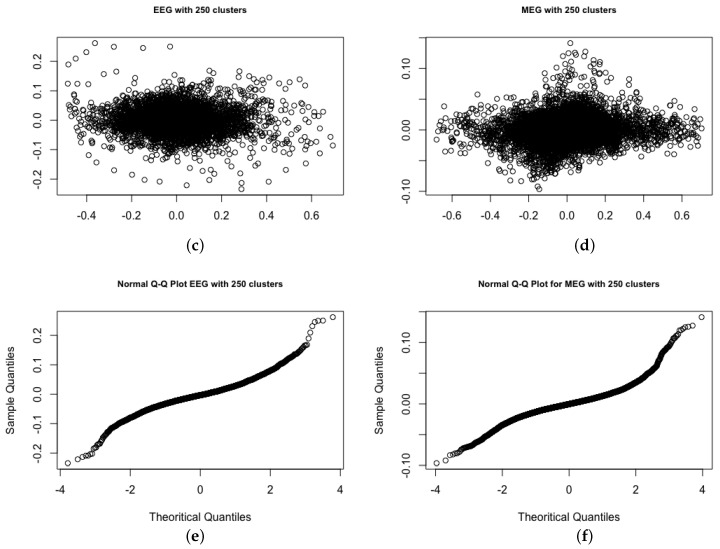
Brain activation for scrambled faces using icm algorithm—residual diagnostics: time series of residuals, (**a**) EEG, (**b**) MEG; residuals versus fitted values, (**c**) EEG, (**d**) MEG; residual normal quantile–quantile plots, (**e**) EEG, (**f**) MEG.

We assessed the goodness of fit of the model by checking the residual time series plot, normal quantile–quantile plot and residuals versus fitted values after running the ACS-ICM and ICM algorithms. This was done by computing the residuals for both EEG and MEG after applying both algorithms. The residuals were computed as ${\hat{\epsilon }}_{M}\left(t\right)=M\left(t\right)-X{}_{M}\hat{S}\left(t\right)$ and ${\hat{\epsilon }}_{E}\left(t\right)=E\left(t\right)-X{}_{E}\hat{S}\left(t\right)$ at each time point t=1,…,T. The assumption made for the residuals was that they should be draws from a mean-zero Gaussian distribution if the assumed model generated the observed data. The residual time series plot for EEG and MEG from the ACS-ICM algorithm is displayed in Figure 10, panels (a) and (b). The plots from Figure 11, panels (a) and (b), also depicts residuals time series plots obtained from ICM for EEG and MEG, respectively. Examining the plots, the residual time series plots obtained from both algorithms exhibit similar patterns for MEG and EEG. However, there are significant improvements seen in estimates from ACS-ICM. Specifically for the EEG data, there are sensors with relatively large peaks remaining from the ICM but significant improvements from ACS-ICM as we observe no fewer residuals patterns relative to ICM. In the case of MEG data, we observe that the residuals obtained from ACS-ICM reveal few sensors with peaks remaining as compared to ICM, where there are more sensors with large peaks and residuals.

In Figure 10 and Figure 11, panels (c) and (d), we show plots of the residuals versus fitted values from ACS-ICM and ICM. For the EEG data, the ACS-ICM residuals reveal fewer extreme values with smaller residual patterns but more outliers are seen in the residuals obtained from ICM comparably. The residuals obtained from ICM are characterized by higher values to the left of zero and lower values to the right of zero. In the case of MEG data, the residuals obtained from ACS-ICM also show fewer extreme values with a smaller residual pattern but a similar resemblance for residuals obtained from the ICM algorithm with few extreme values outside the zero band. We observe more extreme values in the residual plot obtained from ICM than that obtained from ACS-ICM. This signifies improvements of the ACS-ICM algorithm over ICM. Inspecting Figure 10, panels (e) and (f), reveals normal quantile–quantile plots for the EEG and MEG residuals obtained from the ACS-ICM algorithm. There is no deviation from normality observed from the EEG and MEG data. Hence, the Gaussian assumption holds from using the ACS-ICM algorithm. In the case of the ICM, in Figure 11, panels (e) and (f) depict the normal quantile–quantile plots for the EEG and MEG data. In this case we observe a clear divergence from the normal distribution for the EEG and MEG residuals. In particular, we see a strong deviation from normality in the left and right tail of the distribution for the EEG data. There is also a deviation from normality in the right tail of the distribution for the MEG data.

In summary, the residual analysis revealed the use of the ACS-ICM algorithm resulted in estimates with a better fit of the spatial mixture model for the EEG and MEG data relative to ICM. Thus our proposed approach leads to improvements in point estimation and model selection uniformly in all settings in simulation studies and in our application with larger objective function values and improved model fit based on residual analysis.

## 5. Discussion and Conclusions

In this section, we provide numerical results obtained in the data analysis, limitations of the proposed approach and the prospects for future research. We have developed an ACS-ICM algorithm for spatiotemporal modeling of combined MEG/EEG data for solving the neuroelectromagnetic inverse problem. Adopting a Bayesian finite mixture model with a Potts model as a spatial prior, the focus of our work has been to improve source localization estimates, model selection and model fit. The primary contribution is the design and implementation of the ACS-ICM algorithm as an approach for source localization that result in better performance over ICM, which is very positive uniformly in every setting on simulation studies and real data application. Another key development is the technique implemented in choosing the tuning parameters for the ACS-ICM by using an outer level optimization that numerically optimizes the choice of the tuning parameters for this algorithm. This strategy ensures that the optimal tuning parameters based on the data and problem complexity are selected.

### 5.1. Numerical Results

In our simulation studies, we observed four significant improvements associated with ACS-ICM over ICM: (1) ACS-ICM neural source estimates provided improved correlation between estimated and truth sources uniformly across all settings considered; (2) the objective function values obtained from the posterior density values for ACS-ICM were larger than those obtained from ICM uniformly across all settings considered; (3) ACS-ICM showed significant improvement with respect to the total mean square error for all cluster sizes considered compared to ICM; (4) ACS-ICM exhibited improved performance in terms of both bias and mean square error for the non-regular problem of estimating number of mixture components. Moreover, the application of ACS-ICM to real data led to higher quality estimates with larger maximized posterior density values. These improvements have demonstrated the advantage of the ACS-ICM algorithm when compared with ICM in both the face perception analysis as well as the simulation studies. In addition to implementing the ACS-ICM algorithm for point estimation, we demonstrated how a nonparametric bootstrap can be used to obtain standard errors, confidence intervals and T-maps for the proposed methodology. This was done to account for uncertainty in our point estimates of the neural source activity.

### 5.2. Limitations of the Proposed Approach

An important limitation of the simulation studies is the use of white noise added to the signals. This is because MEG/EEG data would have structured noise that arise from, e.g., motion, and such noise would be spatially correlated. The spatially correlated noise will make the simulation scenarios more challenging, which we expect to result in a decline in performance. We did not pursue this scenario in our simulation and we will consider it in our future studies.

### 5.3. Prospects for Future Research

In our current work, we are implementing ACS-ICM for the spatial mixture model developed in [13]. We hope in the future to extend the model by considering a robust error structure in the MEG/EEG model. The model currently assumes that the errors are independent in time. This will be extended by allowing for an autoregressive structure. A second extension would be to relax the assumption that the errors have a Gaussian distribution by incorporating a multivariate t distribution for the error terms. Integrating these extensions, we will develop a new joint model for the MEG/EEG data and implement the ACS-ICM and ICM algorithms for the neuroelectromagnetic inverse problem.

Furthermore, when we obtained the source estimates from the ACS-ICM algorithm, we mapped a function of them (the total power) on the cortex and in that map we used no thresholding. That is to say, the locations were not thresholded so we can see all the locations with estimated power. For our future studies we hope to map the total power on the cortex with a threshold so that we can see the locations with highest power. In a better way to choose the threshold, our next objective is to extend this work by implementing thresholding of cortical maps using random field theory [41]. Random field theory is mainly applied in dealing with thresholding problems encountered in functional imaging. This is used to solve the problem of finding the height threshold for a smooth statistical map, which gives the required family-wise error rate. In going forward with our current work, the idea is to take the point estimate obtained from ACS-ICM and standard errors (obtained from bootstrap) to provide estimates of p-values for t-statistics pertaining to the number of activated voxels comprising a particular region.

It should be noted that the ACS-ICM algorithm and spatial model developed can also be applied to studies involving multiple subjects. Expanding from a single subject model to a model developed for multiple subjects would be of great interest for the MEG/EEG inverse problem. This will be based on developing a fully Bayesian analysis based on a divide and conquer Markov Chain Monte Carlo (MCMC) method [42]. This approach for Bayesian computation with multiple subjects is to partition the data into partitions, perform local inference for each piece separately, and combine the results to obtain a global posterior approximation.

## Figures and Tables

**Table 1:** Simulation study I-Percentage of relative improvement in Total Mean-Squared Error (TMSE) of the neural source estimators decomposed into variance and squared bias from ICM to ACS-ICM. This total was obtained separately for locations in active regions and then for the inactive region.

		Active Region			Inactive Region		
**Algorithm**	**Clusters**	**TMSE (%)**	**(Bias)** ${}^{2}$	**Variance**	**TMSE (%)**	**(Bias)** ${}^{2}$	**Variance**
		**%**	**%**	**%**	**%**	**%**	**%**
	*K* = 2						
ICM→ACS-ICM	250	9.78	11.11	8.93	9.93	6.15	13.16
ICM→ACS-ICM	500	6.63	4.39	8.57	9.48	9.71	9.26
ICM→ACS-ICM	1000	2.95	2.04	4.03	3.86	1.57	5.70
	K=3						
ICM→ACS-ICM	250	5.10	7.80	2.76	4.97	4.31	5.60
ICM→ACS-ICM	500	24.85	25.42	24.31	15.89	18.26	13.61
ICM→ACS-ICM	1000	36.57	53.61	20	10.83	10	11.61
	K=4						
ICM→ACS-ICM	250	12.24	11.11	12.88	8.90	14.19	4.94
ICM→ACS-ICM	500	17.94	14.75	20.71	2.86	3.10	2.64
ICM→ACS-ICM	1000	29.28	30.30	30.65	2.76	3.62	2.06
	K=9						
ICM→ACS-ICM	250	31.14	22.77	27.65	15.44	13.07	17.58
ICM→ACS-ICM	500	14.83	11.40	18.20	17.0	20.39	13.71
ICM→ACS-ICM	1000	23.79	25.52	22.08	8.65	7.03	10.14

**Table 2:** Simulation study II—bias and Mean Square Error (MSE) of estimated number of mixture components ($\hat{K}$) from the 1000 simulation replicates when the algorithms were run with K=10.

	K=2		K=3		K=4		K=9	
**Algorithm**	$\mathrm{Bias}(\hat{K})$	$\mathrm{MSE}(\hat{K})$	$\mathrm{Bias}(\hat{K})$	$\mathrm{MSE}(\hat{K})$	$\mathrm{Bias}(\hat{K})$	$\mathrm{MSE}(\hat{K})$	$\mathrm{Bias}(\hat{K})$	$\mathrm{MSE}(\hat{K})$
	The case where the true signals were well-separated							
ICM	0.11	0.13	0.06	0.42	0.20	0.44	−2.54	6.19
ACS-ICM	0.04	0.06	0.02	0.38	0.10	0.31	−2.01	4.46
	The case where the true signals were less well-separated							
ICM	0.11	0.13	0.525	0.58	−1.02	1.63	−4.83	16.12
ACS-ICM	0.05	0.07	0.35	0.41	−1.00	1.31	−3.68	10.47

**Figure A7:**
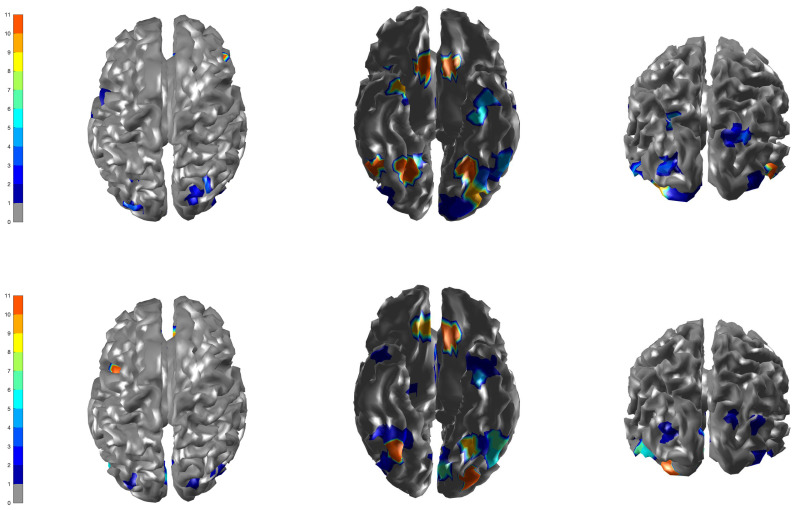
The spatial profile of brain activity from ACS-ICM based on our bootstrap replicates. **Row 1** displays standard deviations of the total power of the estimated source activity; **Row 2** displays the T-map.

## Data Availability

Publicly available datasets were analyzed in this study. This data can be found here https://www.fil.ion.ucl.ac.uk/spm/data/mmfaces (accessed on 26 January 2021).

## Appendix A

**Table A1:** Simulation study I—Average (Ave.) correlation between the neural source estimates and the true values for the ICM and ACS-ICM algorithms. The simulation study is based on R=500 simulation replicates where each replicate involves the simulation of MEG and EEG data based on a known configuration of the neural activity depicted in Appendix A, Figure A1 and Figure A2.

		*K* = 2	*K* = 3	*K* = 4	*K* = 9
**Algorithm**	**Clusters**	**Ave. Corr.**	**Ave. Corr.**	**Ave. Corr.**	**Ave. Corr.**
ICM	250	0.60	0.63	0.62	0.54
ACS-ICM	250	0.64	0.67	0.63	0.59
ICM	500	0.53	0.55	0.49	0.44
ACS-ICM	500	0.56	0.61	0.53	0.46
ICM	1000	0.41	0.43	0.40	0.37
ACS-ICM	1000	0.46	0.47	0.45	0.43

**Table A2:** Simulation study I—Total Mean-Squared Error (TMSE) of the neural source estimators decomposed into variance and squared bias for the ICM and ACS-ICM algorithms. This total was obtained separately for locations in active regions and then for the inactive region.

		Active Region			Inactive Region		
**Algorithm**	**Clusters**	**TMSE**	**(Bias)** ${}^{2}$	**Variance**	**TMSE**	**(Bias)** ${}^{2}$	**Variance**
	K=2						
ICM	250	92	36	56	141	65	76
ACS-ICM	250	83	32	51	127	61	66
ICM	500	196	91	105	211	103	108
ACS-ICM	500	183	87	96	191	93	98
ICM	1000	271	147	124	285	127	158
ACS-ICM	1000	263	144	119	274	125	149
	*K* = 3						
ICM	250	490	237	253	523	255	268
ACS-ICM	250	465	219	246	497	244	253
ICM	500	1203	582	621	705	345	360
ACS-ICM	500	904	434	470	593	282	311
ICM	1000	1657	817	840	674	321	353
ACS-ICM	1000	1051	379	672	601	289	312
	*K* = 4						
ICM	250	776	378	396	674	289	385
ACS-ICM	250	681	336	345	614	248	366
ICM	500	1404	651	753	804	387	417
ACS-ICM	500	1152	555	597	781	375	406
ICM	1000	2493	1100	1393	796	359	437
ACS-ICM	1000	1763	797	966	774	346	428
	*K* = 9						
ICM	250	2100	918	1182	1541	727	814
ACS-ICM	250	1446	709	737	1303	632	671
ICM	500	2515	1246	1269	1260	618	642
ACS-ICM	500	2142	1104	1038	1046	492	554
ICM	1000	3561	1720	1839	1549	740	809
ACS-ICM	1000	2714	1281	1433	1415	688	727

## Appendix B

We present results from the ACS-ICM and ICM algorithms with tuning parameters for the cluster size of 500. The cortical maps displaying the spatial patterns of the total power for estimated sources are represented below.
